# A dual GAN with identity blocks and pancreas-inspired loss for renewable energy optimization

**DOI:** 10.1038/s41598-025-00600-7

**Published:** 2025-05-13

**Authors:** Mostafa Elbaz, Wael Said, Gamal M. Mahmoud, Hanaa Salem Marie

**Affiliations:** 1 Department of Computer Science, Faculty of Computers and Informatics, Kafrelsheikh University, Kafrelsheikh, Egypt; 2 Computer Science Department, Faculty of Computers and Informatics, Zagazig University, Zagazig, 44511 Egypt; 3 Department of Electrical Engineering, Pharos University in Alexandria, Alexandria, Egypt; 4 Faculty of Artificial Intelligence, Delta University for Science and Technology, Gamasa, 35712 Egypt

**Keywords:** Renewable energy, Metaheuristic loss, Generative adversarial networks (GANs), Identity block, Image augmentation, Energy science and technology, Computer science

## Abstract

Integrating energy and solar imagery is essential for electrical engineers in renewable energy prediction, consumption analysis, regression modeling, and fault detection applications. A significant challenge in these areas is the limited availability of high-quality datasets, which can hinder the accuracy of the predictive models. To address this issue, this paper proposes leveraging Generative Adversarial Networks (GANs) to generate synthetic samples for training. Despite their potential, traditional GAN face challenges such as mode collapse, vanishing gradients, and pixel integrity issues. This paper introduces a novel architecture, Penca-GAN, which enhances GANs through three key modifications: (1) dual loss functions to ensure pixel integrity and promote diversity in augmented images, effectively mitigating mode collapse and improving the quality of synthetic data; (2) the integration of an identity block to stabilize training, preserving essential input features and facilitating smoother gradient flow; and (3) a pancreas-inspired metaheuristic loss function that dynamically adapts to variations in training data to maintain pixel coherence and diversity. Extensive experiments on three renewable energy datasets—SKY images, Solar images, and Wind Turbine images—demonstrate the effectiveness of the Penca-GAN architecture. Our comparative analysis revealed that Penca-GAN consistently achieved the lowest Fréchet Inception Distance (FID) scores (164.45 for SKY, 113.54 for Solar, and 109.34 for Wind Turbine), indicating superior image quality compared to other architectures. Additionally, it attains the highest Inception Score (IS) across all datasets, scoring 71.43 for SKY, 87.65 for Solar, and 90.32 for Wind Turbine. Furthermore, the application of Penca-GAN significantly enhanced the fault detection capabilities, achieving accuracy improvements from 85.92 to 90.04% for solar panels and from 86.06 to 90.43% for wind turbines. These results underscore Penca-GAN’s robust performance in generating high-fidelity synthetic images, significantly advancing renewable energy applications, and improving model performance in critical tasks such as fault detection and energy prediction.

## Introduction

In the realm of renewable energy, images play a pivotal role across various tasks, including energy prediction, fault detection, and power consumption management^1–4^. Accurate analysis of solar and other renewable energy imagery enables engineers to optimize energy production, identify anomalies in energy systems, and improve overall efficiency^5–7^. These tasks are essential for advancing sustainable energy solutions and ensuring the reliability of renewable energy systems^8^.

Despite the critical importance of imagery in these applications, a significant limitation is the availability of high-quality datasets. The scarcity of labeled data poses challenges for effectively training machine learning models^9,10^. As a result, many researchers and practitioners are hindered in developing robust solutions that can be generalized well in real-world scenarios. This limitation underscores the need for innovative approaches to augment existing datasets and enhance model performance^11^.

GANs have emerged as a powerful tool for data augmentation, enabling the generation of synthetic images that can complement limited datasets. By training on existing data and generating new, realistic samples, GANs can help improve the robustness and accuracy of models used in renewable energy applications. This capability is particularly valuable in instances where data collection is expensive or time-consuming^12–14^.

However, GANs are not without their challenges. Issues such as mode collapse, where the generator produces a limited variety of outputs, and pixel integrity, which refers to maintaining the quality and coherence of the generated images, are significant obstacles. These problems can adversely affect the performance of models that rely on augmented data, leading to suboptimal results in critical applications like fault detection and energy prediction^15–17^. To address these challenges, our approach leverages a novel metaheuristic method to maintain pixel integrity in augmented images. Drawing inspiration from biological systems, specifically the behavior of the pancreas, a new loss function is introduced to promote pixel coherence while enhancing the diversity of the generated samples.

This methodology integrates seamlessly with our GAN architecture, which includes an identity block to stabilize training and ensure consistency in the generated outputs. Mode collapse is a critical issue that often arises during GAN training. This occurs when the generator produces a limited set of outputs, failing to capture the full diversity of the training data. This phenomenon restricts the variability of the generated samples and compromises the overall performance of the models that depend on this synthetic data for training. In the context of renewable energy applications, mode collapse can lead to insufficient representations of diverse scenarios, ultimately affecting tasks such as fault detection and energy prediction.

To mitigate the effects of mode collapse, our proposed architecture incorporates an identity block within the GAN framework. This identity block is a stabilizing mechanism that allows the network to maintain the essential features of the input data while facilitating a smoother gradient flow during training. By preserving the key characteristics of the generated images, the identity block enhances the generator’s ability to produce a more diverse range of outputs. This approach helps combat mode collapse and contributes to improved pixel integrity, ensuring that the augmented images are both varied and coherent. As a result, the inclusion of the identity block plays a vital role in enhancing our GAN architecture’s overall robustness and effectiveness in renewable energy applications.

The proposed methodology introduces a pancreas-inspired metaheuristic loss function to address the challenge of maintaining pixel integrity in the generated images. This approach draws inspiration from the pancreatic regulatory mechanisms, which maintain homeostasis by adjusting insulin levels to control blood sugar.

In biological systems, the pancreas functions as a feedback control system. When blood sugar levels rise, the pancreas releases insulin, which facilitates glucose uptake by cells, thereby lowering blood sugar levels. Conversely, when blood sugar drops, insulin secretion decreases, allowing for glucose release into the bloodstream. This dynamic modulation ensures that blood sugar levels remain within a healthy range, demonstrating a self-regulating mechanism that continuously adapts to changes in the internal environment.

In the context of generative modeling, the generator in a GAN can be viewed as analogous to the pancreas. Its goal is to produce high-quality images that accurately represent the underlying data distribution. However, during training, the generator may struggle with mode collapse, where it produces a limited variety of outputs. This lack of diversity is akin to a failure in the pancreas’ ability to regulate insulin levels effectively, leading to imbalances in output.

The pancreas-inspired metaheuristic loss function stands apart from other biological-inspired optimization techniques, such as genetic algorithms and swarm intelligence, by employing a dynamic feedback mechanism that continuously adjusts to the generator’s performance during training. While traditional approaches often focus on population-based strategies, which evolve solutions over generations without real-time adaptability, the pancreas-inspired method emphasizes maintaining pixel-level integrity and diversity in generated images through immediate corrective actions. This not only fosters a more responsive adaptation to the training dynamics but also ensures that the outputs remain coherent and high-quality. Moreover, unlike many biological metaheuristics that optimize a singular objective, the pancreas-inspired approach integrates multiple facets of image generation—specifically, pixel integrity and diversity—into a holistic framework. This comprehensive strategy allows for a more nuanced optimization process, ultimately enhancing the robustness and effectiveness of the generative model in complex tasks like image synthesis.

This study investigates the potential of GANs and data augmentation techniques to enhance energy output prediction models in renewable energy resources (RER). The goal is to demonstrate how these innovations can transcend existing technological limits, achieving superior benchmarks in performance, variance, and reliability in industry applications. Key distinctions arise in the implementation of these features compared to current methodologies, particularly in the sensitivity of generative models to architectural design and parameter optimization. The proposed modifications are significant enough that, without them, generating models of equivalent caliber would be nearly impossible. While both augmentations are critical for success, challenges in convergence persist, necessitating careful consideration of their integration. This study aims to showcase the effectiveness of the redesigned model with these enhancements, emphasizing the contributions of the novel loss function and the overall advancements relative to existing technologies.

The contribution points are as follows:

1. Development of a new metaheuristic loss function inspired by the pancreas, focusing on maintaining pixel integrity while promoting diversity in augmented images.
2. Introduced a new dual loss function based on a new metaheuristic loss function inspired by the pancreas, which focuses on maintaining pixel integrity while promoting diversity in augmented images.
3. Implementing strategies within the GAN architecture to effectively mitigate mode collapse, thereby ensuring a broader representation of data in generated samples and demonstrating improved pixel integrity in augmented images, contributes to higher fidelity in output critical for tasks such as fault detection and energy prediction.
4. The novel architecture of the GAN based on the dual loss function outperforms the different architectures of GANs in mode collapse mitigation, image diversity, and generated images.
5. The new GAN architecture based on the dual loss function helps different architectures improve their performance in the segmentation and detection process. It may be used in the future in wide applications in the real world, especially in renewable energy.
6. There is evidence of enhanced accuracy and reliability in the detection tasks when using the generated images, thereby supporting the methodology’s practical application in renewable energy systems.

Novelty aspects of the pancreas-inspired loss function and how it differs from other biological techniques:

1. Pancreas-Inspired Metaheuristic Function: Introduction of a novel loss function inspired by the intelligent behavior of the pancreas, specifically its regulatory mechanisms in maintaining homeostasis. This approach emphasizes the importance of maintaining pixel integrity while promoting diversity in the generated images, setting it apart from traditional techniques.
2. Dynamic Adaptation: Unlike other biological techniques that may rely on fixed rules or patterns, the pancreas-inspired function adapts to the variations in the training data, allowing for more responsive adjustments during the GAN training process. This adaptability enhances the generator’s ability to produce more output.
3. Focus on Integrity and Diversity: While many biological-inspired methods prioritize either integrity or diversity, the proposed pancreas-inspired function effectively balances both objectives. The proposed dual focus helps resolve common data augmentation issues, such as mode collapse and pixel coherence, offering a more holistic solution.
4. Biological Relevance: The pancreas was chosen as an inspiration because of its critical role in regulating biological processes through feedback mechanisms. This relevance provides a unique framework not typically explored in existing GAN methodologies, often drawing inspiration from more straightforward biological concepts.
5. Enhanced Learning Efficiency: Our approach mimics the pancreas’ ability to manage complex interactions, allowing for more efficient learning and the generation of high-quality images. This contrasts with other biological techniques that may not effectively capture such dynamic interactions, leading to less optimal training outcomes.

This paper is organized into several key sections to provide a comprehensive understanding of our proposed methodology and its implications for renewable energy applications. Following this introduction, Sect. 2 reviews the relevant literature on GANs, highlighting existing challenges such as mode collapse and dataset limitations. Section 3 details the architecture of the proposed Penca-GAN, including the integration of the identity block and the pancreas-inspired metaheuristic loss function. Section 4 presents the experimental setup, including the datasets used and the evaluation metrics employed to assess the performance of our model. In Sect. 5, The experimental results are discussed, demonstrating the effectiveness of the proposed approach in enhancing image diversity and integrity. Finally, Sect. 6 concludes the paper, summarizing the key findings and offering suggestions for future research directions. This structured approach ensures that readers can easily follow the progression of our research and understand the significance of our contributions to the field.

## Related work

Generative Adversarial Networks (GANs) have emerged as a pivotal technology for image augmentation, offering a powerful framework for synthesizing high-quality, realistic data. The foundational work by Brophy et al.^18^ established the adversarial training paradigm, where a generator and discriminator are trained in tandem, inspiring a plethora of subsequent advancements. This adversarial setup has proven to be highly effective in addressing diverse challenges within image generation and augmentation.

The versatility of GANs has led to their widespread adoption across various domains, including image synthesis, style transfer, and data augmentation^19,20^. Moreover, GANs are increasingly utilized in cybersecurity applications, such as intrusion detection, steganography, password cracking, and anomaly detection, demonstrating their potential in addressing evolving security challenges^21–24^.

To enhance the controllability and specificity of the generated outputs, Conditional GANs (cGANs)^25^were introduced. By conditioning the generation process on auxiliary information, such as class labels, cGANs enable targeted attribute generation, significantly increasing the relevance of synthetic data in various tasks. In the realm of image-to-image translation, Pix2Pix^26^leveraged a cGAN architecture to transform images from one domain to another, relying on paired training data. However, the reliance on paired data limited its applicability in scenarios with scarce paired datasets. CycleGAN^27^ addressed this limitation by enabling unpaired image-to-image translation, facilitating transformations across domains without requiring corresponding image pairs.

Advancements in high-resolution image generation and style control were realized with the introduction of StyleGAN^28^and its successors. These models allow for the disentanglement of high-level attributes and style information, producing highly detailed and realistic images. Complementing this, super-resolution GANs (SRGANs)^29^focus on generating high-resolution images from low-resolution inputs, augmenting datasets with enhanced imagery. Further advancements in image super-resolution have been highlighted in recent studies^30–33^, demonstrating the transformative potential of GAN-based frameworks for various imaging applications.

For improved interpretability and feature manipulation, InfoGAN^34^maximizes the mutual information between the generated images and latent variables, enabling structured and interpretable outputs. Augmented GANs (AugGANs)^35,36^ integrate data augmentation techniques directly into the GAN architecture, enhancing training and sample diversity.

Addressing training instability, Wasserstein GANs (WGANs)^37^utilize the Wasserstein distance as a loss function, providing a more stable training environment. WGAN-GP builds upon this by incorporating a gradient penalty to enforce Lipschitz continuity. Self-Attention GANs (SAGANs)^38^introduce self-attention mechanisms to capture long-range dependencies, while Progressive Growing GANs (ProGANs)^39^employ a progressive training strategy to improve stability and quality. Boundary Equilibrium GANs (BEGANs)^40^ balance generator and discriminator training for enhanced image quality and diversity.

Specialized applications of GANs include Semi-Supervised GANs, which leverage both labeled and unlabeled data for high-quality generation, and Cycle Consistency GANs, which enforce consistency across different domains. Colorful Image GANs (CiGANs) focus on colorizing grayscale images, serving as a valuable augmentation tool.

Despite these advancements, several research gaps remain. Many models, such as Pix2Pix and CycleGAN, rely on large-scale datasets, limiting their applicability in data-scarce domains. Interpretability and controllability of the generated outputs remain challenges, with latent space complexity hindering user control. Training stability continues to be a concern, requiring robust training techniques. There is also a need for GANs that can learn from weakly supervised or noisy data, produce diverse outputs, and seamlessly integrate multiple augmentation techniques.

The Self-Attention GAN (SAGAN)^38^introduces self-attention mechanisms, allowing the model to capture long-range dependencies within images, which results in more coherent and diverse outputs. Similarly, the Progressive Growing GAN (ProGAN)^39^ uses a progressive training strategy that gradually increases the complexity of the generated images, significantly improving both stability and quality during training.

In cases where labeled data is scarce, the Semi-Supervised GAN leverages labeled and unlabeled data, enabling the generation of high-quality samples while effectively using the limited labeled data available. The Cycle Consistency GAN emphasizes the importance of consistency across different domains, reinforcing the reliability of the generated images.

The Boundary Equilibrium GAN (BEGAN)^40^ introduces a boundary equilibrium approach that effectively balances the generator and discriminator training, resulting in enhanced quality and diversity of the generated images. For specialized applications, the Colorful Image GAN (CiGAN) focuses on generating color images from grayscale inputs, serving as a valuable augmentation tool for datasets that lack color information. Table 1 shows recent research on GAN based approaches for renewable energy applications and their limitations.

**Table 1:** Recent research on GAN-Based approaches for renewable energy applications.

Research	Dataset	Methods Used	Results	Limitations
Fathallah et al.^41^	CelebA dataset for face synthesis with 200 K celebrity photos Stacked MNIST dataset for mode collapse assessment	Identity blocks added to the architecture Modified loss function and label smoothing implemented	IGAN outperforms other GAN models regarding IS and FID. IGAN converges to a real data distribution faster and produces more stable, high-quality images.	Mode collapse due to the vanishing gradient problem Difficulty in generating high-quality images with fewer dataset images
Schreiber et al.^42^	Two wind datasets Two solar datasets	Comparative study of the Wasserstein distance, binary-cross-entropy Loss, and Gaussian copula Evaluation of the generated power distribution and terrain-specific distributions	The Wasserstein GAN is superior in modeling temporal and spatial relations and power distribution. GANs can model terrain-specific power distributions with limited data.	Limited studies on how well GAN model power distribution Limited data compared with previous studies
Li et al.^43^	Real-time series data for wind and solar power. External universal meteorological features are used for scenario generation.	Improved VAEGAN model with the spectral normalization technique. Controllable vector using mutual information maximization.	Improved controllable generation of renewable energy scenarios. Better performance in generating various statistical features.	–
Hu et al.^44^	Historical wind power generation data were used. Multiple sets of generated wind power scenarios were created.	GANs for scenario generation. Considers the spatial correlation of wind power generation.	Generates medium- and long-term wind power scenarios. Captures randomness and spatial correlation in wind generation.	–
Feng et al.^45^	Renewable energy output data • Corresponding predicted data	Conditional Spectral Normalization Generative Adversarial Networks (CSNGAN) Markov chain-based scenario generation method	The CSNGAN method accurately describes the uncertainty of the renewable energy output. Outperforms the Markov chain-based scenario generation method.	–
Jiang et al.^46^	Wind data from 20 sites in Washington State. Solar data from 32 sites in Washington State.	Unsupervised distribution learning method based on the GAN Stochastic constrained optimization for scenario forecasts	The generated trajectories reflect future power generation dynamics. Captures the temporal, spatial, and fluctuant characteristics of real power generation processes.	Uncertainty in the timing, magnitude, and duration of fluctuations. Local optima challenges in nonconvex adversarial networks.

### Research gap

There is a lack of literature focused on the following research aspects:

1. GANs such as cGAN, fcGAN, DCGAN, and RNN-based GANs are rare to report in renewable energy applications. There is a lack of mathematical demonstration and analytical research discussion regarding a new labeled GAN architecture-based approach in the field of renewable energy in complex optimization with Machine Learning (ML) and the Internet of Things (IoT) interconnection scenario. Currently, no popular method covers and compares these renewable energy problems, which considers a new labeled GAN architecture applied for ML and IoT interconnections.
2. Industrial engineering system optimization, particularly renewable energy solutions, is still in its infancy. Existing methods and technologies are few or completely unavailable, leading to a lack of comparisons. Researchers have not yet explored validating the developed technology with several case studies or real-time practical scenarios. Most of the current work does not address the compatibility constraints of the input training power systems.

According to the state of the art in the overall literature, the GAN technique, especially the recent advanced deep GANs, is a very effective method to learn a generative data distribution model. This aspect is well used in different applications. However, reporting in renewable energy applications such as droop parameters, Model predictive Control realization in renewable generation solutions, and predictive-variable-time-step is rare, particularly in the IoT. The latest review papers on IoT-based power energy systems did not explore these modern methods. From the above section, it was stated that GAN works on four fundamentals and requires more and more samples, essentially driven by one optimization algorithm’s output solution. As the recently claimed best optimization optimizer, the Panca metaheuristic also has aspects of handling such huge-sized power-based variable dimensions; its GAN-based optimization properties are not yet well investigated. Thus, all the above-discussed reviewed methods have some cons and are insufficient to solve and optimize the two problems. Hence, all the stated industrial issues and fields will be addressed in this manuscript.

### Research questions

The core of the problem and the research questions of the study should be framed in the context of existing literature to ensure the positioning of the identified gap. The search revealed that the novelty in the proposed Penca-GAN lies in two key aspects: the formulation of the loss function using the system identification model for a discrete range of density and the development of the network architecture incorporating the identity block as the generator for image smoothing.

This research explicitly states the study’s central research question: how can a Dual-GAN with Identity Blocks and Pancreas-Inspired Loss improve the optimization of renewable energy? Generating power scenarios is crucial for renewable energy systems; however, current practices rely on static models. Dynamic models require access to real-time data and are typically proprietary, limiting their broad application. The proposed study addresses these challenges by developing a publicly accessible Dual-GAN that uses historical net power data to simulate future power scenarios.

Through Penca-GAN, advancements to the state of the art are made in two respects. Consequently, the following research questions are formulated:

Research ***Question 1***: Does the proposed Penca-GAN have the efficacy to solve for the optimized parameters of the honeycomb design representing the use of solar energy in Jeddah, Saudi Arabia? If any, how does the AI-based approach compare to benchmark optimization techniques? Research ***Question 2***: Can the performance of the proposed Penca-GAN be generalized to modeling curb appeal? How does Penca-GAN fare compare to state-of-the-art GAN and deep learning models?

## Methodology

The proposed methodology for discriminating between real and fake images consists of several key components working together to achieve the desired objective. At the system’s core is the Discriminator module, which is responsible for classifying input images as either genuine or forged. The system employs two distinct input sources to provide the discriminator with a diverse and comprehensive set of training data. The first is the Energy-Image Generator, which takes the original input image and generates a series of “energy images” that capture various visual features and characteristics of the image. These energy images represent the multifaceted input, allowing the discriminator to learn from a richer set of image representations. Complementing the real image data, the system also includes a Fake Images block, which generates synthetic or manipulated images designed to mimic the properties of the genuine images. By exposing the discriminator to real and fake samples during training, the system can ensure that the model learns to identify the subtle yet critical differences between authentic and forged visual data. To further enhance the discriminator’s ability to discern real from fake, the methodology incorporates a specialized loss function known as the Penca-loss function for checking pixel integrity Loss II. This loss function evaluates the consistency and plausibility of the individual pixels within the input images, providing a granular assessment of the image authenticity. By minimizing this Penca-loss function, the system can guide the discriminator to prioritize the detection of pixel-level manipulations, which are often indicative of image forgery.

The overall Loss I block combines the Penca-loss function with other relevant metrics, such as classification accuracy or adversarial Loss, to create a comprehensive loss function that the system aims to minimize during training. This holistic loss function allows the discriminator to optimize its performance in distinguishing between real and fake images, leveraging the various sources of information and feedback provided by the different components of the methodology. By integrating the Energy-Image Generator, Fake Images, Discriminator, Penca-loss function, and the overall Loss I, the proposed methodology creates a powerful and nuanced system capable of accurately identifying forged or manipulated images, with potential applications in areas such as image authentication, quality control, and digital forensics.

Figure 2 shows the block diagram of the methodology. The first component is the Energy-Image Generator. This module takes the input image and generates a set of “energy images” that capture the original image’s different visual features and characteristics. The energy images represent the input image that highlights specific aspects, such as textures, edges, or color distributions. By generating these energy images, the system can extract a more comprehensive set of features from the data input, which can aid in the discrimination between real and fake images. Next, the Fake Images block is responsible for generating synthetic or manipulated images that mimic the characteristics of the real images. These fake images are created using various techniques, such as GANs or other image synthesis methods. The purpose of including these fake images in the training process is to expose the discriminator model to a diverse set of real and artificial examples so that it can learn to distinguish between them effectively.

The discriminator is the core component of the system. This module takes both real and fake images as inputs and is trained to classify them as either genuine or forged. Through the training process, the discriminator learns to identify the subtle differences between real and fake images, developing a robust understanding of the visual cues and characteristics that distinguish authentic images from manipulated ones.

The Penca-loss function for checking pixel integrity Loss II is a specialized loss function that measures the integrity and consistency of individual pixels within the images. This loss function helps the system assess the images’ fidelity and authenticity by evaluating the pixel-level information’s coherence and plausibility. By minimizing this Loss, the system can better identify instances where the pixel-level characteristics have been altered or manipulated, which is a key indicator of a fake or forged image.

The Loss I block represents the overall loss function that the system minimizes during training. This loss function likely combines the Penca-loss function with other relevant metrics, such as classification accuracy or adversarial Loss, to comprehensively assess the system’s performance. The goal is to train the Discriminator model to minimize this Loss, ultimately enhancing its ability to distinguish between real and fake images. The Real Sample block represents the authentic, real-world images used as input to the system and the fake images generated earlier. These real samples serve as the ground truth for the training process, allowing the discriminator to learn the characteristics and patterns of the genuine images. The “+” block combines the Penca-loss function and the real sample to generate the overall Loss I, which the system tries to minimize during training. This integration of the loss function and the real sample data allows the system to effectively optimize the discriminator’s performance in distinguishing between real and fake images.

**Fig. 1:**
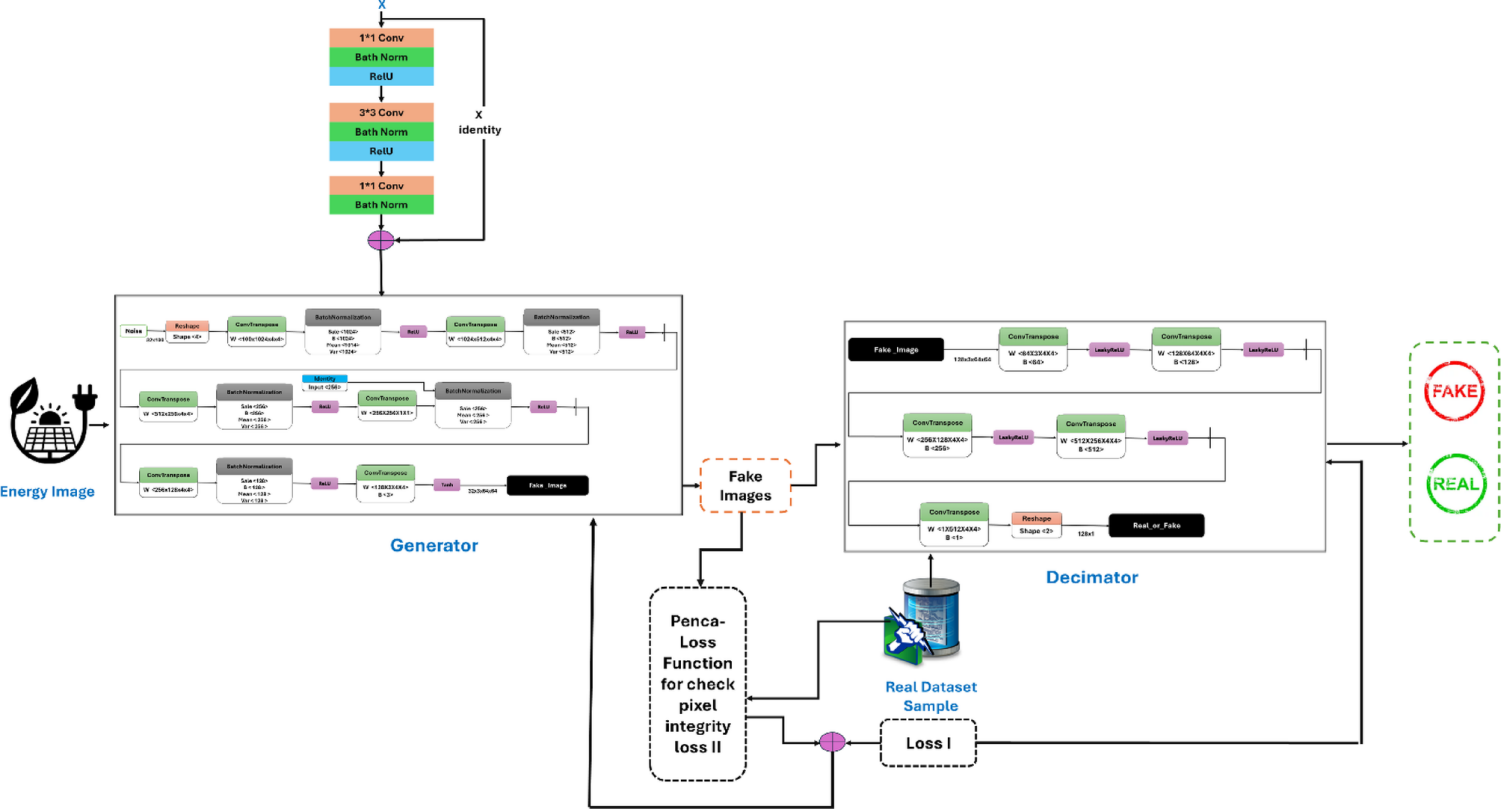
Methodology block diagram.

### Dataset description

**Fig. 2:**
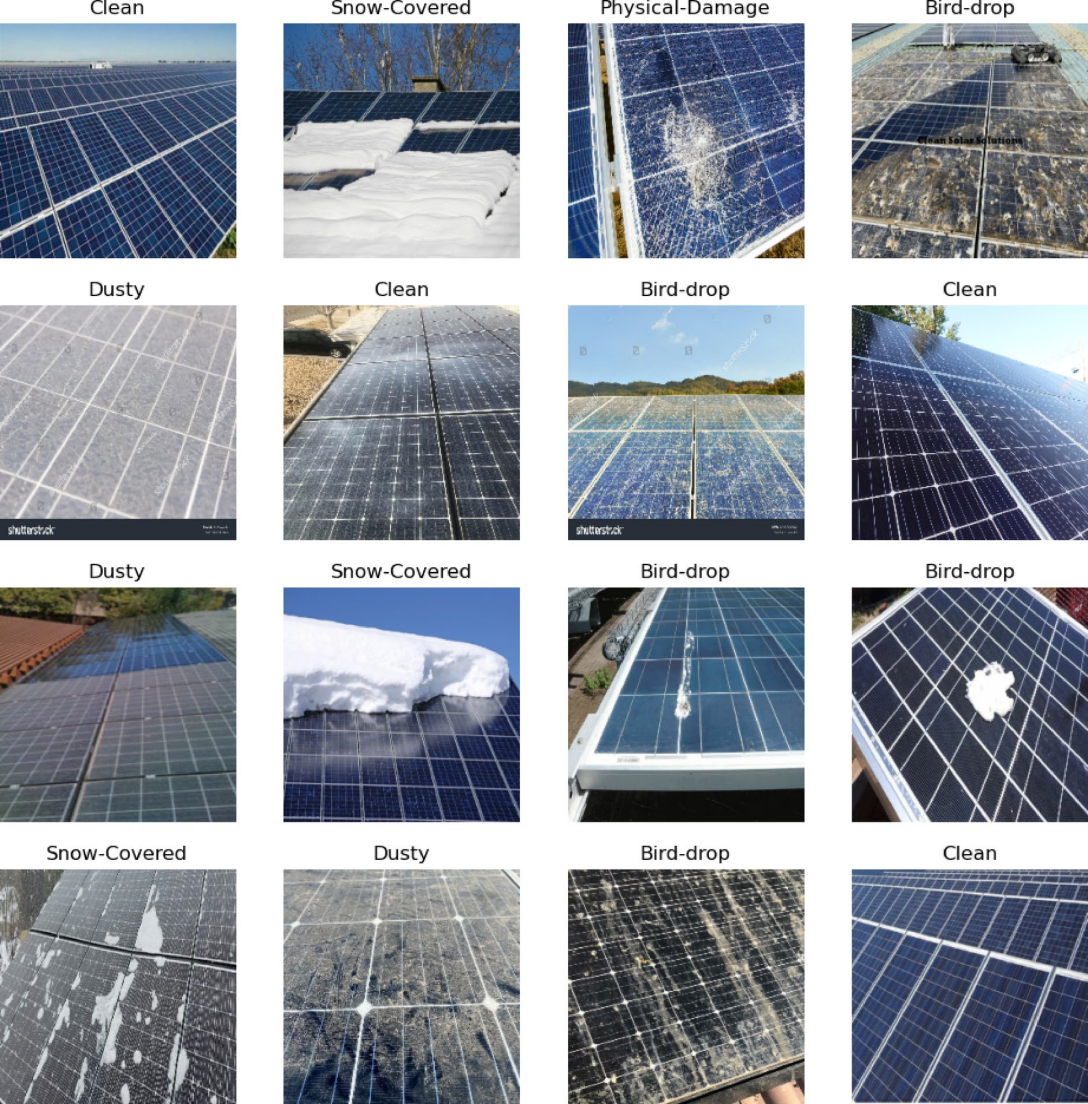
Samples of solar panel faults dataset.

The methodology uses three distinct datasets to enhance the robustness and applicability of the image discrimination system. The first dataset focuses on solar panels^47^, comprising a diverse collection of images showcasing various solar panel installations in real-world environments. This dataset is categorized into six distinct classes: Physical Damage, Electrical Damage, Snow Cover, Cleanliness, Dust Accumulation, and Bird Droppings. Each class contains 69 images, resulting in a total of 414 images, all captured at a resolution of 1024 × 768 pixels. The acquisition process involved capturing images in various environments, including residential rooftops, commercial solar farms, and remote locations, under different weather conditions such as sunny, cloudy, and rainy, ensuring a comprehensive dataset. The class distribution is balanced, with each category represented equally, which is crucial for reducing potential bias in the classification model. The preprocessing steps included normalization of pixel values to a standard range, as well as augmentation techniques to enhance model robustness. The dataset was split into training (70%), validation (15%), and test (15%) sets, ensuring effective model evaluation and training. Figure 2 show samples of dataset.

The second dataset consists of sky images^48^, specifically curated to represent solar radiation conditions relevant to solar energy generation. This dataset encompasses high-resolution images (1920 × 1080 pixels) depicting various atmospheric phenomena, categorized into six classes: Clear Skies, Partly Cloudy, Overcast, Sunny, Cloudy with Sunbeams, and Stormy Weather. Each class contains 100 images, leading to a total of 600 images. Images were captured across various geographic locations at different times of the day and under various weather conditions to ensure comprehensive representation of sky states. The class distribution is as follows: 100 images for Clear Skies, 100 for Partly Cloudy, 100 for Overcast, 100 for Sunny, 100 for Cloudy with Sunbeams, and 100 for Stormy Weather. Preprocessing steps involved resizing for uniformity and color normalization to adjust for variations in lighting conditions. Additional augmentation techniques, such as brightness adjustment and contrast enhancement, were applied to improve model training effectiveness. By analyzing these images, the system can better assess the authenticity of solar panel images and their relevance to solar radiation data.

**Fig. 3:**
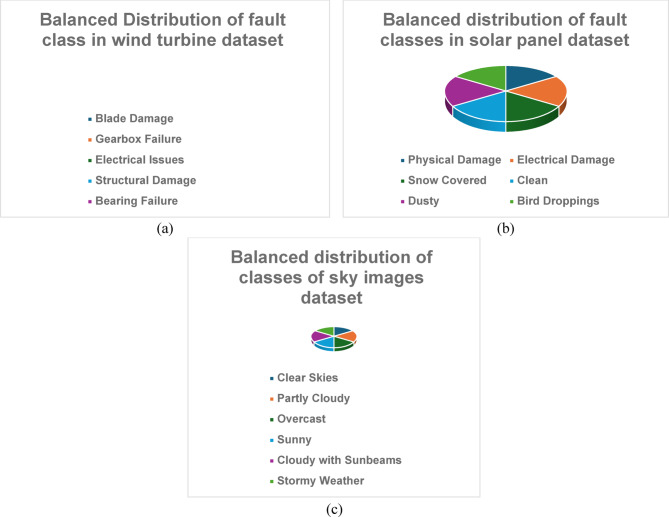
Distribution of classes for datasets used.

The third dataset focuses on wind turbines^49^, featuring images illustrating both onshore and offshore wind turbine setups. This dataset includes six distinct classes representing common fault types: Blade Damage, Gearbox Failure, Electrical Issues, Structural Damage, Bearing Failure, and Control System Malfunction. Each class contains 150 images, resulting in a total of 900 images captured at a resolution of 1280 × 720 pixels. Images were taken during various operational scenarios, including normal operation and fault conditions, captured during different times of the day and under various weather conditions to reflect real-world variability. The class distribution includes 150 images for each fault type, ensuring that the model is exposed to a balanced representation of each category. Preprocessing steps included noise reduction and contrast adjustment to enhance image clarity, alongside augmentation techniques such as rotation and cropping to artificially increase the dataset size. These three datasets provide a rich and varied foundation for training the image discrimination system, ensuring its effectiveness in distinguishing real from fake images across different renewable energy contexts. This comprehensive approach ultimately contributes to improved monitoring and management of solar and wind energy resources, with the availability of these datasets clearly stated to facilitate reproducibility and further research efforts. Figure 3 shows the distribution of each class for the three different datasets.

### GANs with an identity block

In the proposed methodology, the Generative Adversarial Network (GAN) plays a crucial role in generating and processing energy images, particularly by incorporating an identity block. This architecture enhances the model’s ability to maintain important data characteristics while producing high-quality synthetic images.

The GAN consists of two primary components: The Generator and the Discriminator. The Generator is responsible for creating synthetic images, while the discriminator evaluates the authenticity of both the real and generated images. In this methodology, the Generator is specifically designed to produce energy images that capture the essential features of the original input images, such as textures, patterns, and relevant visual information. The identity block within the Generator serves as a critical mechanism for preserving the identity of the input image during the transformation process. This block allows the Generator to retain key features and spatial relationships, ensuring that the generated energy images closely resemble the original images. By incorporating skip connections, the identity block facilitates the flow of information, enabling the model to effectively combine low- and high-level features. This architecture helps to mitigate the risk of losing important details that could aid in the discriminator’s ability to distinguish between real and fake images.

The processing of the energy images begins with the input image being passed through the Generator. As the image is transformed into an energy representation, the identity block ensures that essential features are preserved while enhancing other visual aspects relevant to the task. The resulting energy images are then fed into the discriminator, which evaluates their authenticity alongside real images from the training datasets.

The GAN architecture employed in this methodology is designed to effectively generate energy images while preserving the essential features from the input data. This architecture consists of two primary components: The Generator and the Discriminator, each serving distinct but complementary roles in image generation and discrimination. The Generator is structured to transform input images into energy images that encapsulate key visual features. Central to this design is the inclusion of an identity block, which employs skip connections to facilitate the seamless flow of information between layers. This allows the Generator to retain critical details from the original input while enhancing other features relevant to the task. By maintaining the identity of the input image, the Generator ensures that the generated energy images closely resemble their authentic counterparts, thereby improving the quality and reliability of the synthetic outputs.

Multiple convolutional layers are employed in the Generator to extract features from the input images. These layers progressively downslope the image, capturing a range of textures and patterns. The identity block intervenes at various points in the architecture, enabling the model to combine low-level features with higher-level abstractions. This results in energy images that reflect the original image’s characteristics and emphasize the relevant visual information necessary for effective discrimination.

The discriminator, on the other hand, evaluates the authenticity of both the real and generated images. It employs a series of convolutional layers that progressively downslope the input images, extracting hierarchical features that help distinguish genuine images from synthetic ones. The discriminator’s architecture is designed to be robust, allowing it to learn the subtle differences between real and fake images effectively. It outputs a probability score indicating whether an input image is real or generated.

Together, the Generator and Discriminator create a dynamic adversarial training process. The Generator learns to produce increasingly realistic energy images, while the discriminator continuously improves its ability to detect forgeries. This interplay fosters a cycle of improvement, where both components enhance each other’s performance over time. The architecture is optimized through a comprehensive loss function that combines the Penca-loss for pixel integrity with other relevant metrics, ensuring that the system focuses on generating high-quality images and maintaining their authenticity. Overall, with its identity block, this GAN architecture provides a robust framework for generating and processing energy images in the context of renewable energy applications.

**Fig. 4:**
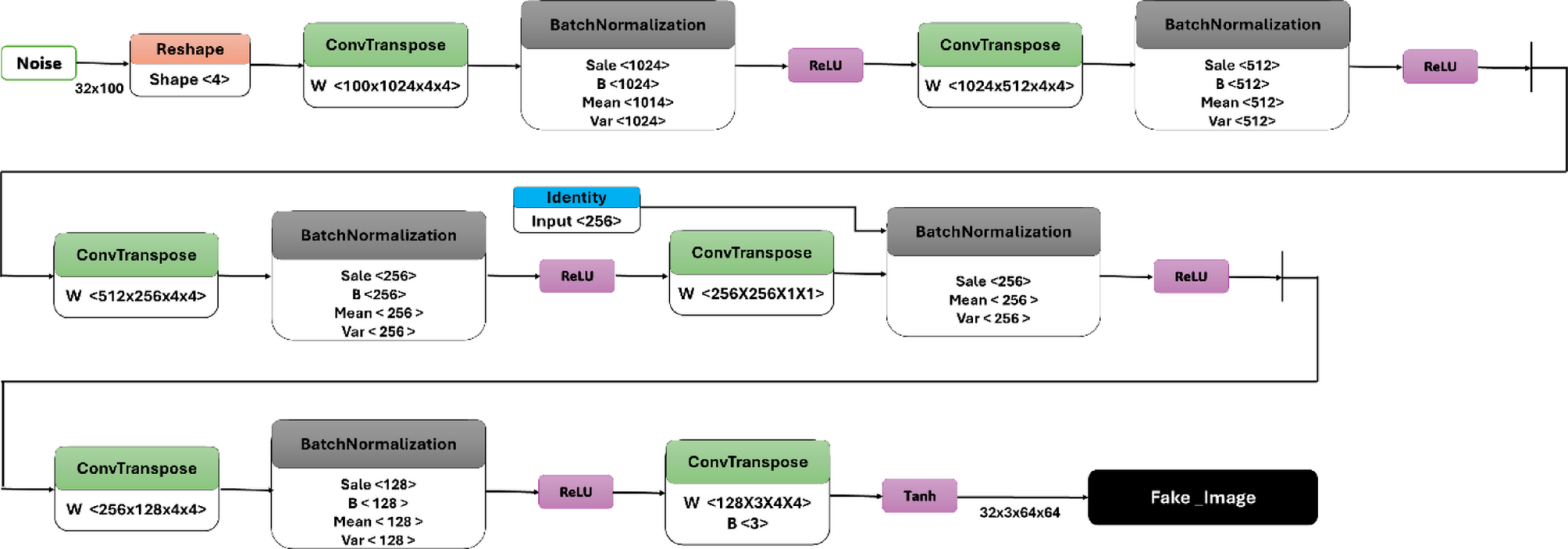
IGAN generator architecture.

**Fig. 5:**
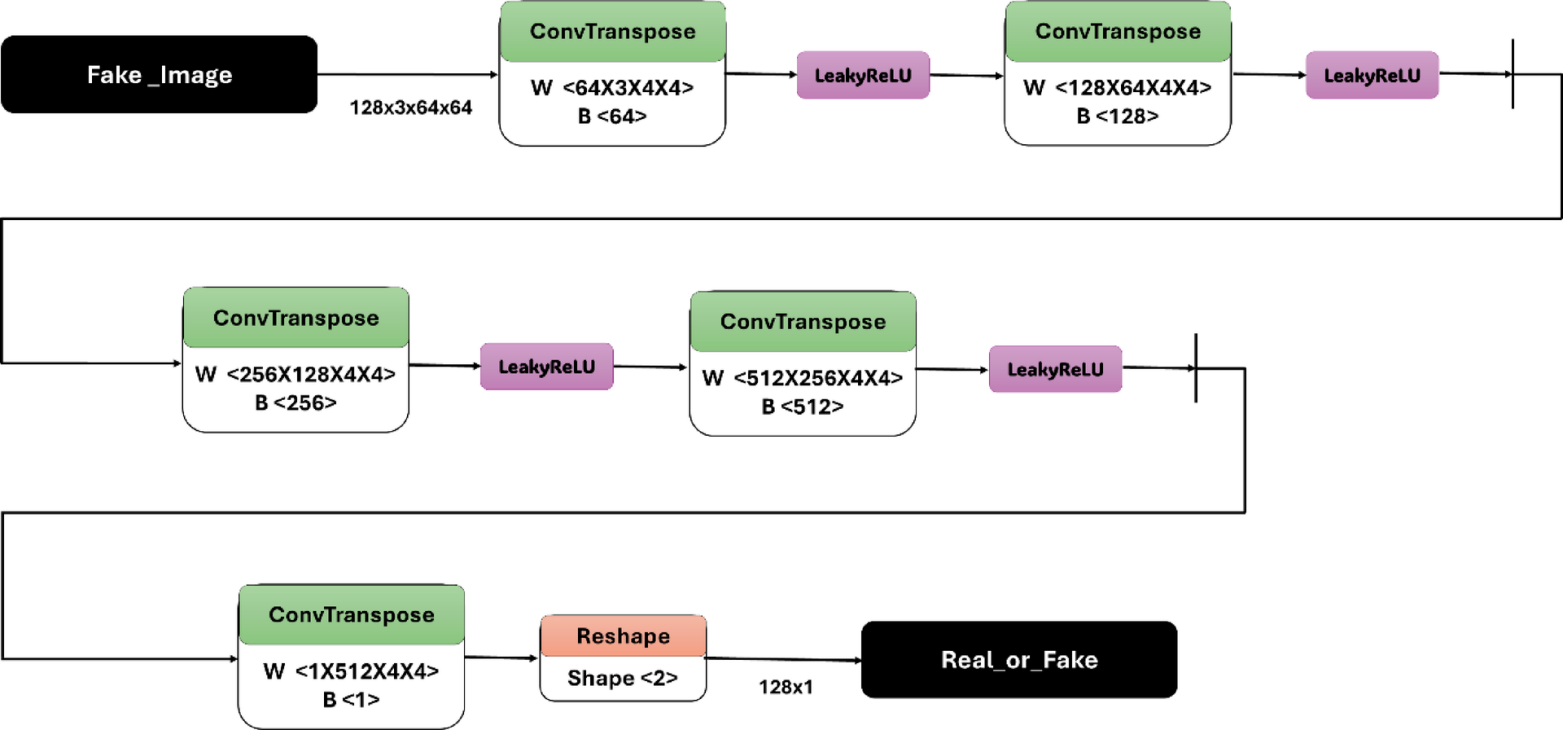
IGAN discriminator architecture.

The architecture of the IGAN generator, as detailed in Table 2, consists of a sequence of layers designed to transform noise input into a high-resolution image. The initial layer reshapes a noise vector of size 100 × 1 × 1 into a tensor of size 4 × 1 × 1. This is followed by a series of transposed convolution layers, which progressively increase the spatial dimensions of the feature maps while reducing the number of channels. For instance, the first transposed convolution layer expands the tensor to 1024 × 4 × 4, using learnable weights W. Batch normalization and ReLU activation are applied to stabilize training and introduce non-linearity, respectively. This pattern continues through multiple layers, with the output size gradually increasing to 3 × 64 × 64, corresponding to the final generated image. The last layer employs a Tanh activation function to produce pixel values in the range of [− 1,1], resulting in the final output labeled Fake_Image. The architecture is visually represented in Fig. 4, which illustrates the data flow through the generator. Conversely, the architecture of the IGAN discriminator, outlined in Table 3, operates as a binary classifier to distinguish between real and generated images. The input to the discriminator is either a real image or a fake image of size 3 × 64 × 64. The model employs a series of convolutional layers, starting with a transposed convolution that reduces the spatial dimensions while increasing the depth of the feature maps. Notably, Leaky ReLU activations are used after each convolutional layer to allow for a small, non-zero gradient when the unit is not active. The architecture concludes with a reshaping layer that transforms the output into a binary classification, indicating whether the input image is real or fake. This dual architecture approach, combining generator and discriminator networks, is crucial for the effective training of GANs and enhances the model’s ability to generate high-quality images, as depicted in Fig. 5.

**Table 2:** The architecture of IGAN generator.

Layer	Type	Input Size	Output Size	Parameters
1	Noise + Reshape	100 × 1 × 1	4 × 1 × 1	–
2	ConvTranspose	–	1024 × 4 × 4	W < 100 × 1024 × 4 × 4>
3	BatchNormalization	1024 × 4 × 4	1024 × 4 × 4	Scale < 1024>, B < 1024>
4	ReLU	1024 × 4 × 4	1024 × 4 × 4	–
5	ConvTranspose	1024 × 4 × 4	512 × 8 × 8	W < 1024 × 512 × 4 × 4>
6	BatchNormalization	512 × 8 × 8	512 × 8 × 8	Scale < 512>, B < 512>
7	ReLU	512 × 8 × 8	512 × 8 × 8	–
8	ConvTranspose	512 × 8 × 8	256 × 16 × 16	W < 512 × 256 × 4 × 4>
9	BatchNormalization	256 × 16 × 16	256 × 16 × 16	Scale < 256>, B < 256>
10	ReLU	256 × 16 × 16	256 × 16 × 16	–
11	ConvTranspose	256 × 16 × 16	256 × 32 × 32	W < 256 × 256 × 1 × 1>
12	BatchNormalization	256 × 32 × 32	256 × 32 × 32	Scale < 256>, B < 256>
13	ReLU	256 × 32 × 32	256 × 32 × 32	–
14	ConvTranspose	256 × 32 × 32	128 × 64 × 64	W < 256 × 128 × 4 × 4>
15	BatchNormalization	128 × 64 × 64	128 × 64 × 64	Scale < 128>, B < 128>
16	ReLU	128 × 64 × 64	128 × 64 × 64	–
17	ConvTranspose	128 × 64 × 64	3 × 64 × 64	W < 128 × 3 × 4 × 4>
18	Tanh	3 × 64 × 64	3 × 64 × 64	–
Output	Fake_Image	–	3 × 64 × 64	–

**Table 3:** The architecture of IGAN discriminator.

Layer	Type	Input Size	Output Size	Parameters
1	Input (Real or Fake_Image)	3 × 64 × 64	3 × 64 × 64	–
2	ConvTranspose	3 × 64 × 64	64 × 32 × 32	W < 64 × 3 × 4 × 4>, B < 64>
3	LeakyReLU	64 × 32 × 32	64 × 32 × 32	–
4	ConvTranspose	64 × 32 × 32	128 × 16 × 16	W < 128 × 64 × 4 × 4>, B < 128>
5	LeakyReLU	128 × 16 × 16	128 × 16 × 16	–
6	ConvTranspose	128 × 16 × 16	256 × 8 × 8	W < 256 × 128 × 4 × 4>, B < 256>
7	LeakyReLU	256 × 8 × 8	256 × 8 × 8	–
8	ConvTranspose	256 × 8 × 8	512 × 4 × 4	W < 512 × 256 × 4 × 4>, B < 512>
9	LeakyReLU	512 × 4 × 4	512 × 4 × 4	–
10	ConvTranspose	512 × 4 × 4	1 × 1 × 1	W < 1 × 512 × 4 × 4>, B < 1>
11	Reshape	1 × 1 × 1	2 (binary)	–
Output	Real_or_Fake	–	2 (real/fake)	–

#### Architecture of the identity block

The Identity Block is critical in the proposed GAN methodology for renewable energy applications. This architectural component is designed to help maintain the essential features and characteristics of the input images during the generation process, contributing to the overall stability and quality of the synthetic samples.

The Identity Block comprises a repeating pattern of three key layers: a 1 × 1 convolutional layer, a batch normalization layer, and a ReLU activation function. This combination allows the model to preserve important spatial and contextual information from the input while also applying non-linear transformations to enhance the generator’s learning capabilities.

The proposed methodology can address common issues such as mode collapse and pixel integrity by integrating the identity block within the GAN architecture. The Identity Block acts as a stabilizing mechanism, ensuring that the generated images retain the visual characteristics and diversity required for effective training and deployment in renewable energy tasks. The strategic placement of the Identity Block allows the generator to learn a more robust and coherent mapping from the input to the output space. This, in turn, improves the fidelity of the generated samples, making them more suitable for downstream applications like fault detection, energy prediction, and consumption analysis.

### Pancreas-inspired meta heuristics loss function

The proposed methodology introduces a novel pancreas-inspired metaheuristic loss function to address the challenge of maintaining pixel integrity in the generated images. This unique approach draws inspiration from the pancreas’s intelligent behavior, which regulates insulin levels to maintain homeostasis in the human body.

Similar to how the pancreas adjusts insulin production to keep blood sugar levels within a healthy range, the pancreas-inspired loss function aims to preserve the coherence and consistency of the generated pixels in the synthetic images. By modeling this biological regulatory mechanism, the loss function encourages the generator to produce samples that exhibit a harmonious balance of pixel-level attributes, ensuring that the generated images remain visually coherent and realistic.

The key insight behind this biologically inspired approach is the recognition that the pancreas operates as an intelligent, self-regulating system that continuously monitors and adapts its output to maintain a stable internal environment. The generator in the GAN architecture can be viewed as an analogous system responsible for producing high-quality images that faithfully represent the underlying data distribution. Just as the pancreas uses feedback loops to modulate insulin secretion, the pancreas-inspired loss function provides a similar control mechanism to guide the generator toward generating images with pixel-level integrity.

Incorporating this metaheuristic loss function into the overall GAN framework addresses common issues such as mode collapse and pixel degradation. By incentivizing the generator to preserve the spatial and contextual relationships between pixels, the loss function helps ensure that the generated samples are diverse and maintain a high degree of visual fidelity. This, in turn, enhances the utility of the synthetic data for downstream applications in the renewable energy domain, where tasks like fault detection and energy prediction rely heavily on the quality and consistency of the input imagery.

The unique aspect of this pancreas-inspired approach lies in its ability to leverage biological principles to tackle the challenges inherent to generative modeling. Drawing inspiration from the regulatory mechanisms observed in natural systems, the proposed methodology introduces a novel and effective way to maintain pixel integrity, ultimately improving the overall performance and robustness of the GAN architecture in renewable energy applications. Algorithm 1 shows the main steps of the pancreas-inspired meta-heuristic loss function.

The Penca-GAN model establishes a profound biomimetic relationship with pancreatic function through its novel loss mechanism. In biological systems, the pancreas maintains glucose homeostasis via a complex feedback loop where beta cells continuously monitor blood glucose concentrations and modulate insulin secretion accordingly. This adaptive regulatory system exhibits remarkable precision in maintaining optimal physiological parameters despite environmental fluctuations. Penca-GAN’s loss function mathematically emulates this biological process by implementing a dynamic regulatory mechanism that monitors pixel-level coherence across generated images. Just as pancreatic beta cells increase insulin production when blood glucose rises above optimal thresholds, the Penca-GAN loss function applies stronger corrective penalties when pixel distributions deviate from natural image statistics. Conversely, when pixel relationships maintain coherence (analogous to normal glucose levels), the regulatory pressure decreases. This biomimetic approach enables a form of “pixel homeostasis” where the generator learns to maintain balanced spatial and contextual relationships between neighboring pixels. The pancreatic insulin regulation system employs multiple signaling pathways with varying temporal dynamics - some responding rapidly to acute changes while others modulate long-term adaptive responses. Similarly, Penca-GAN integrates multi-scale feedback mechanisms operating at different hierarchical levels of the image structure, from local pixel neighborhoods to global compositional elements, mirroring the hierarchical regulatory networks found in biological pancreatic function.

The Pixel Integrity Loss ($\:{\mathbf{L}}_{\mathbf{i}\mathbf{n}\mathbf{t}\mathbf{e}\mathbf{g}\mathbf{r}\mathbf{i}\mathbf{t}\mathbf{y}}$) ensures that the generated images maintain consistency with the real images at the pixel level. If the difference between the real and generated pixel exceeds a threshold $\:\varvec{\upepsilon\:}$, a penalty is applied for each pixel as shown in Eq. (1). Where N is the total number of pixels, $\:\varDelta\:{\varvec{p}}_{\varvec{i}}$ is the pixel difference between the real and generated images at pixel $\:i$, $\:[\varDelta\:{\varvec{p}}_{\varvec{i}}>\varvec{\upepsilon\:}]$ is an indicator function that applies the penalty only if ​ $\:\varDelta\:{\varvec{p}}_{\varvec{i}}\text{}$ exceeds the threshold $\:\varvec{\upepsilon\:}$ and $\:\mathbf{i}\mathbf{n}\mathbf{s}\mathbf{u}\mathbf{l}\mathbf{i}{\mathbf{n}}_{\mathbf{r}\mathbf{a}\mathbf{t}\mathbf{e}}$ is a dynamic factor controlling the penalty, analogous to insulin regulation in the pancreas.

1 $$\:{\mathbf{L}}_{\mathbf{i}\mathbf{n}\mathbf{t}\mathbf{e}\mathbf{g}\mathbf{r}\mathbf{i}\mathbf{t}\mathbf{y}}\mathbf{}=\sum\:_{\mathbf{i}=1}^{\mathbf{N}}(\mathbf{i}\mathbf{n}\mathbf{s}\mathbf{u}\mathbf{l}\mathbf{i}{\mathbf{n}}_{\mathbf{r}\mathbf{a}\mathbf{t}\mathbf{e}}\times\:{\left(\varDelta\:{\varvec{p}}_{\varvec{i}}\right)}^{2}\times\:\:\mathbf{I}[\varDelta\:{\varvec{p}}_{\varvec{i}}>\varvec{\upepsilon\:}]$$

Diversity Loss encourages the model to generate diverse images, reducing the risk of mode collapse by comparing the variance of the generated images to a target diversity level as mentioned in the Eq. (2), where the $\:{\mathbf{L}}_{\mathbf{d}\mathbf{i}\mathbf{v}\mathbf{e}\mathbf{r}\mathbf{s}\mathbf{i}\mathbf{t}\mathbf{y}}\mathbf{}$​ represents the diversity loss, which measures how well the generated images meet the specified target diversity level. The use of $\:\mathbf{m}\mathbf{a}\mathbf{x}\left(0,.\right)\:$function ensures that the diversity loss remains non-negative; if the computed value is negative, indicating that the generated images already meet or exceed the target diversity, the loss is set to zero and t$\:\left(\mathbf{g}\mathbf{e}\mathbf{n}\mathbf{e}\mathbf{r}\mathbf{a}\mathbf{t}\mathbf{e}{\mathbf{d}}_{\mathbf{i}\mathbf{m}\mathbf{a}\mathbf{g}\mathbf{e}\mathbf{s}}\right)$is the variance across the pixel values of the generated images, and the diversity target is the target diversity level. The term $\:\mathbf{d}\mathbf{i}\mathbf{v}\mathbf{e}\mathbf{r}\mathbf{s}\mathbf{i}\mathbf{t}{\mathbf{y}}_{\mathbf{t}\mathbf{a}\mathbf{r}\mathbf{g}\mathbf{e}\mathbf{t}}\:$signifies the desired level of diversity that the generated images should achieve, serving as a benchmark for evaluation. Loss is positive only when the variance is less than this target, encouraging more diverse outputs.

2 $$\:{\mathbf{L}}_{\mathbf{d}\mathbf{i}\mathbf{v}\mathbf{e}\mathbf{r}\mathbf{s}\mathbf{i}\mathbf{t}\mathbf{y}}\mathbf{}=\mathbf{m}\mathbf{a}\mathbf{x}\left(0,\mathbf{d}\mathbf{i}\mathbf{v}\mathbf{e}\mathbf{r}\mathbf{s}\mathbf{i}\mathbf{t}{\mathbf{y}}_{\mathbf{t}\mathbf{a}\mathbf{r}\mathbf{g}\mathbf{e}\mathbf{t}}-\mathbf{V}\mathbf{a}\mathbf{r}\left(\mathbf{g}\mathbf{e}\mathbf{n}\mathbf{e}\mathbf{r}\mathbf{a}\mathbf{t}\mathbf{e}{\mathbf{d}}_{\mathbf{i}\mathbf{m}\mathbf{a}\mathbf{g}\mathbf{e}\mathbf{s}}\right)\right)$$

The total fitness function is the sum of the negative pixel integrity and diversity loss. The model’s goal is to minimize this fitness function.

3 $$\:\mathbf{F}\mathbf{i}\mathbf{t}\mathbf{n}\mathbf{e}\mathbf{s}\mathbf{s}=-\left({\mathbf{L}\mathbf{}}_{\mathbf{i}\mathbf{n}\mathbf{t}\mathbf{e}\mathbf{g}\mathbf{r}\mathbf{i}\mathbf{t}\mathbf{y}}+{\mathbf{L}}_{\mathbf{d}\mathbf{i}\mathbf{v}\mathbf{e}\mathbf{r}\mathbf{s}\mathbf{i}\mathbf{t}\mathbf{y}}\mathbf{}\right)$$

Penca-GAN distinguishes itself as an efficient generative adversarial network by leveraging a novel metaheuristic inspired by the pancreas, enhancing training stability and output diversity. Unlike traditional metaheuristic algorithms that often focus solely on optimizing a specific objective function, Penca-GAN incorporates a holistic approach that addresses multiple facets of image generation. This includes maintaining pixel integrity while simultaneously promoting diversity in the generated samples. The unique design of the Penca-GAN facilitates the effective mitigation of mode collapse, a common challenge in standard GAN implementations. By integrating these elements, Penca-GAN achieves superior image quality and demonstrates robustness across varied datasets, making it a more versatile and effective solution compared to other metaheuristic-inspired algorithms. Its empirical validation through comprehensive performance metrics further underscores its capabilities, setting a new benchmark in generative modeling.

**Figure:**
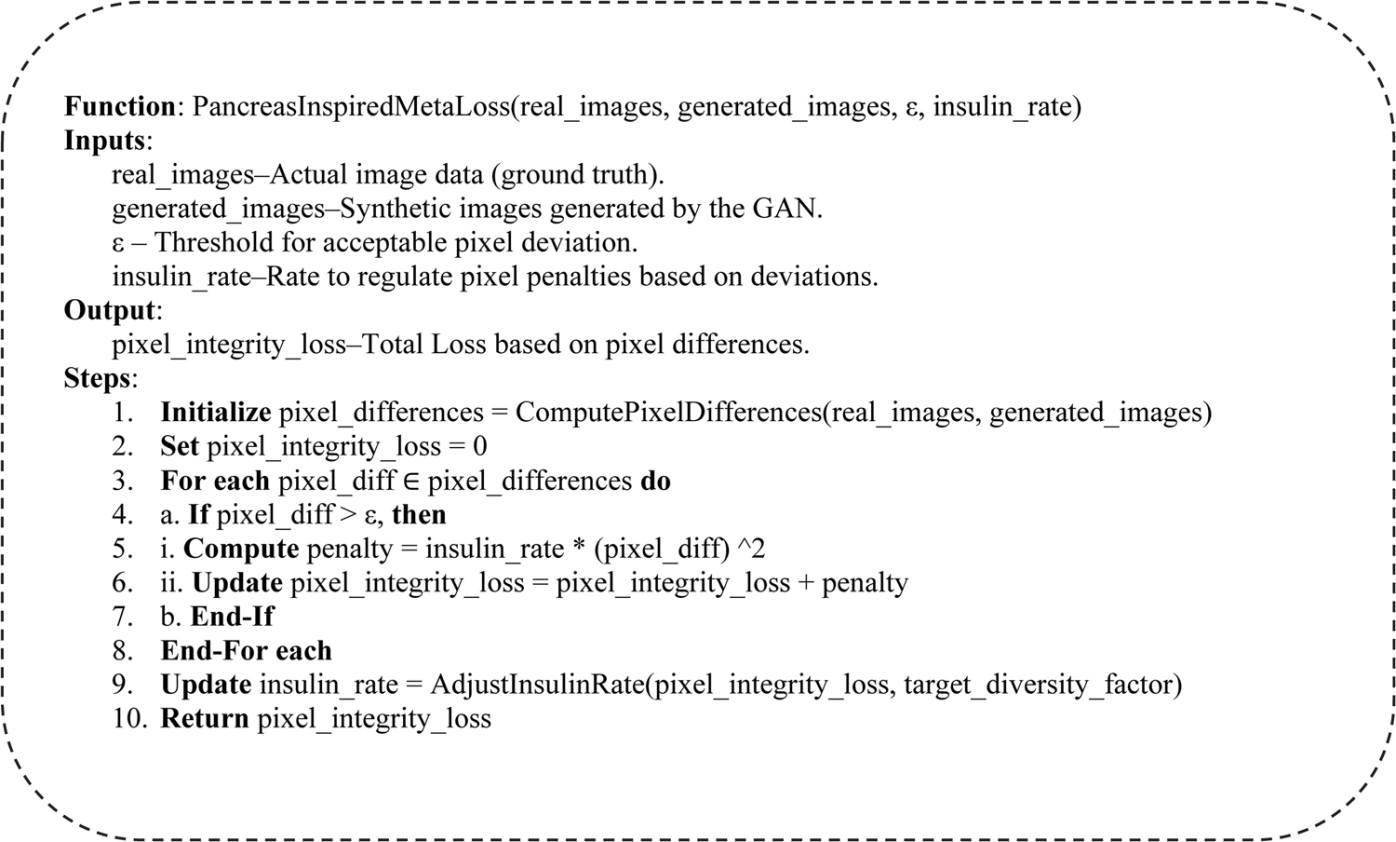
**Algorithm 1:** Main steps of the pancreas-inspired meta heuristics loss function

## Experimental results and discussion

The experimental evaluation employs three distinct datasets: sky, solar panel, and wind turbine images. Our proposed model’s performance is compared against seven well-established GAN architectures, namely: Wasserstein GAN (WGAN), Cycle-GAN, StyleGAN, Conditional GAN (cGAN), Progressive GAN (ProGAN), Self-Attention GAN (SAGAN), 8-connected Pixel Identity GAN with Neutrosophic (ECP-IGANN), GAN with identity blocks inspired by menstrual cycle behavior for missing pixel imputation (MCI-GAN), GAN-based sperm-inspired pixel imputation (GSIP), StyleGAN, Latent Diffusion Models (LDM). and Augmented GAN (AugGAN). This section provides a comprehensive comparison of the image generation quality, model stability, and overall performance across the different architectures. Additionally, the effectiveness of mode collapse mitigation is assessed for each of the seven GAN models on the datasets, demonstrating how the proposed model addresses this common issue in generative tasks across diverse domains.

Ablation study was conducted to investigate the contributions of the individual components of our proposed model. This study isolated and evaluated the impact of various model features on performance across the three datasets: sky, solar panel, and wind turbine images.

The experimental results also present a case study for detecting fault damage in solar panels after and before applying our architecture using six new detection models, such as Vision Transformers (ViT)^50^, Mobilenet-v3^51^, YOLO-v6^52^, Inception-v3^53^, Resnet-101^54^, SPF-Net^55^, and VGG-16^56^. The case study also introduces another case study for wind turbine fault detection.

### Diversity of the augmented images

This subsection of the paper compares our architecture and the seven other GAN architectures in terms of the diversity of the augmented images using FID and IS, using Eqs. (4) and (5), respectively^57,58^. the term $\:{\mu\:}_{r}$ represents the mean of the real images’ feature representations, while $\:{\mu\:}_{g}$ denotes the mean of the generated images’ feature representations. The expression $\:{\mid\:\mid\:{\mu\:}_{r}-{\mu\:}_{g}\mid\:\mid\:}^{2}$ calculates the squared Euclidean distance between these two means, providing a measure of how closely the generated images resemble the real images. The term$\:{\varSigma\:}_{r}$r​ is the covariance matrix of the real images, and$\:{\varSigma\:}_{g}$ is the covariance matrix of the generated images. The expression $\:2{\left({\varSigma\:}_{r}{\varSigma\:}_{g}\right)}^{1/2}$ for the interaction between the distributions of the real and generated images. The parameter $\:{T}_{r}$​ is a scaling factor that adjusts the contribution of the covariance difference to the overall distance metric. Together, these components quantify the similarity between the distributions of the real and generated images.

The notation $\:{Ex}_{\sim\:G}\:$​ indicates the expectation to take over the distribution of the generated images G. The term $\:{D}_{KL}\left(p\left(y \mid x\right)\parallel\:p\left(y\right)\right)$ represents the Kullback-Leibler divergence between the conditional label distribution p(y∣x), which indicates the probability of the label y given an image xxx, and the marginal distribution p(y), which represents the overall probability of the labels. This divergence measures the amount of information lost when approximating the true distribution p(y) with p(y∣x). The exponential function exp (⋅) transforms the expected Kullback-Leibler divergence into a score that reflects both the diversity and quality of the generated images, with higher scores indicating better performance. Together, these equations provide valuable metrics for evaluating the quality and diversity of the generated images in generative models.

4 $$\:FID={\mid\:\mid\:{\mu\:}_{r}-{\mu\:}_{g}\mid\:\mid\:}^{2}+{T}_{r}\left({\varSigma\:}_{r}+{\varSigma\:}_{g}-2{\left({\varSigma\:}_{r}{\varSigma\:}_{g}\right)}^{1/2}\right)$$

5 $$\:IS\left(G\right)=\text{e}\text{x}\text{p}\left({Ex}_{\sim\:G}\left[{D}_{KL}\left(p\left(y \mid x\right)\parallel\:p\left(y\right)\right)\right]\right)$$

**Fig. 6:**
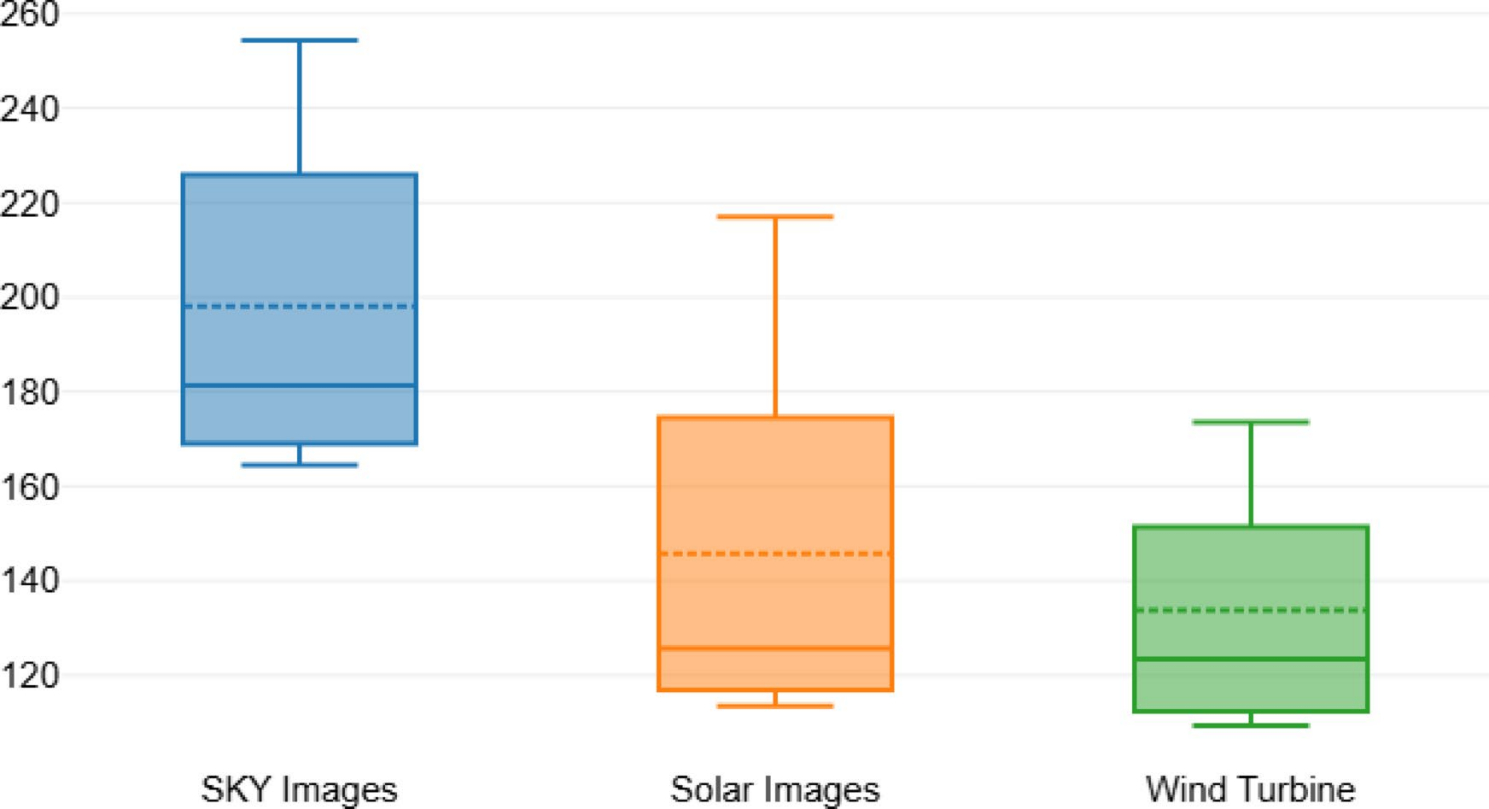
Performance comparison of GAN architectures across different datasets in terms of FID.

The comparison of various GANs based on their Fréchet Inception Distance (FID) scores across three different datasets is detailed in Table 4. The results indicate that Penca-GAN achieved the lowest FID scores across all datasets, with values of 164.45 for sky images, 113.54 for solar images, and 109.34 for wind turbine images. This underscores Penca-GAN’s effectiveness in generating high-fidelity synthetic imagery, as lower FID scores indicate closer alignment with the real data distribution. In contrast, other models such as cGAN and SAGAN exhibited the highest FID scores, with values of 254.37 for solar images and 243.43 for sky images, respectively, suggesting significant shortcomings in their ability to generate realistic images for these datasets. The variation in performance is notable, particularly with traditional models such as AugGAN and ProGAN, which also showed higher FID scores, indicating greater distances from the real data distributions. Figure 6 visually illustrates these performance comparisons, emphasizing the superior performance of the Penca-GAN in generating images across all evaluated categories. The trend in the data clearly demonstrates the advancements in GAN architecture, with newer models like Penca-GAN significantly outperforming their predecessors in terms of image quality and realism.

The hyperparameters used in various Image Augmentation GANs are critical for optimizing performance across different architectures. For example, AugGAN is configured with a learning rate of 0.0002 and a batch size of 16–32, utilizing the Adam optimizer with specific beta values (β₁=0.5, β₂=0.999) and an adversarial loss combined with task-specific objectives. This model features a latent space dimension of 100–256 and is trained for 200 epochs, employing augmentation techniques such as rotation, flipping, and color jittering. In contrast, SAGAN operates with a lower learning rate of 0.0001 and a larger batch size of 32–64, leveraging hinge loss and self-attention modules to enhance feature representation, also trained for 200 epochs. ProGAN employs a decreasing learning rate starting at 0.001 and uses progressive growing, allowing for training on lower-resolution images before gradually increasing resolution, while cGAN is designed with a learning rate of 0.0002 and a batch size of 64–128, focusing on binary cross-entropy and L1 loss functions, also trained for 200 epochs. StyleGAN features a learning rate of 0.001, with a more complex architecture that includes a mapping network and synthesis network, trained with 25,000 images per resolution. LDM (Latent Diffusion) is unique with its very low learning rate range of 0.00001 to 0.0001, a batch size of 32–256, and a significantly higher training duration of 1 million to 2 million steps, emphasizing its thorough training process. Penca-GAN stands out with its perceptual and content-aware loss functions, focusing on pencil-style transformations, and is trained with a learning rate of 0.0001 and a batch size of 24 for 200 epochs. Each model’s specific hyperparameters reflect a tailored approach to balancing learning efficiency, stability, and the quality of the generated images, ultimately contributing to advancements in image augmentation through GANs. Penca-GAN achieves promising results compared to ECP-IGANN^59^, MCI-GAN^60^, GSIP-GAN^61^ etc.

**Table 4:** Fid-based comparison of GAN architectures across three different datasets.

Model	Dataset		
	SKY images	Solar images	Wind turbine
AugGAN	225.98	180.34	164.45
SAGAN	243.43	200.74	167.54
ProGAN	237.65	174.54	151.34
cGAN	254.37	216.92	173.53
StyleGAN	216.32	154.56	149.43
Cycle-GAN	200.43	137.56	129.87
WGAN	180.43	123.54	119.33
Style-GAN	181.34	125.65	123.45
ECP-IGANN	168.98	115.76	112.34
MCI-GAN	165.65	115.09	111.67
GSIP-GAN	169.98	119.98	117.08
LDM	166.31	116.87	110.12
Penca-GAN	164.45	113.54	109.34

The performance of the generative models can be further analyzed through the descriptive statistics of the Fréchet Inception Distance (FID) scores presented in Table 5. The mean FID scores indicate that sky images had the highest average score at 198.1, suggesting a greater distance from the real data distribution compared to the other image types. Solar images followed with a mean FID score of 145.78, while wind turbine images had the lowest mean at 133.81. The standard deviations for these scores reflect variability, with solar images showing the highest variability (35.98) and wind turbine images the lowest (24.03). The range of scores also highlights significant disparities, particularly in sky images (89.92) versus wind turbine images (64.19), indicating that the generative models struggled more with accurately producing sky imagery.

Table 6 provides the results of the Bonferroni post hoc tests applied to the FID scores, revealing significant differences between the image types. The mean differences indicate that sky images had a notably higher FID score compared to both solar images (52.33) and wind turbine images (64.29), with p-values less than 0.001, underscoring the challenges in generating realistic sky images. The comparison between the solar and wind turbine images showed a smaller but statistically significant difference (11.97) with a p-value of 0.021. The confidence intervals for these differences reinforce the findings, indicating that the variations in the FID scores are not only statistically significant but also meaningful in assessing the quality of the generated images across the evaluated datasets.

**Table 5:** Descriptive statistics of the FID score.

	SKY images	Solar images	Wind turbine
Mean	198.1	145.78	133.81
Std. Deviation	33.33	35.98	24.03
Minimum	164.45	113.54	109.34
Maximum	254.37	216.92	173.53
Range	89.92	103.38	64.19
Mean ± Std.	198.1 ± 33.33	145.78 ± 35.98	133.81 ± 24.03

**Table 6:** Bonferroni Post-hoc-Tests of FID score.

		Mean diff.	Std. Error	*P*	95% CI lower limit	95% CI upper limit	
SKY images	Solar images	52.33	2.159	24.236	< 0.001	47.62	57.03
SKY images	Wind turbine	64.29	3.041	21.143	< 0.001	57.67	70.92
Solar images	Wind turbine	11.97	3.683	3.25	0.021	3.94	19.99

**Fig. 7:**
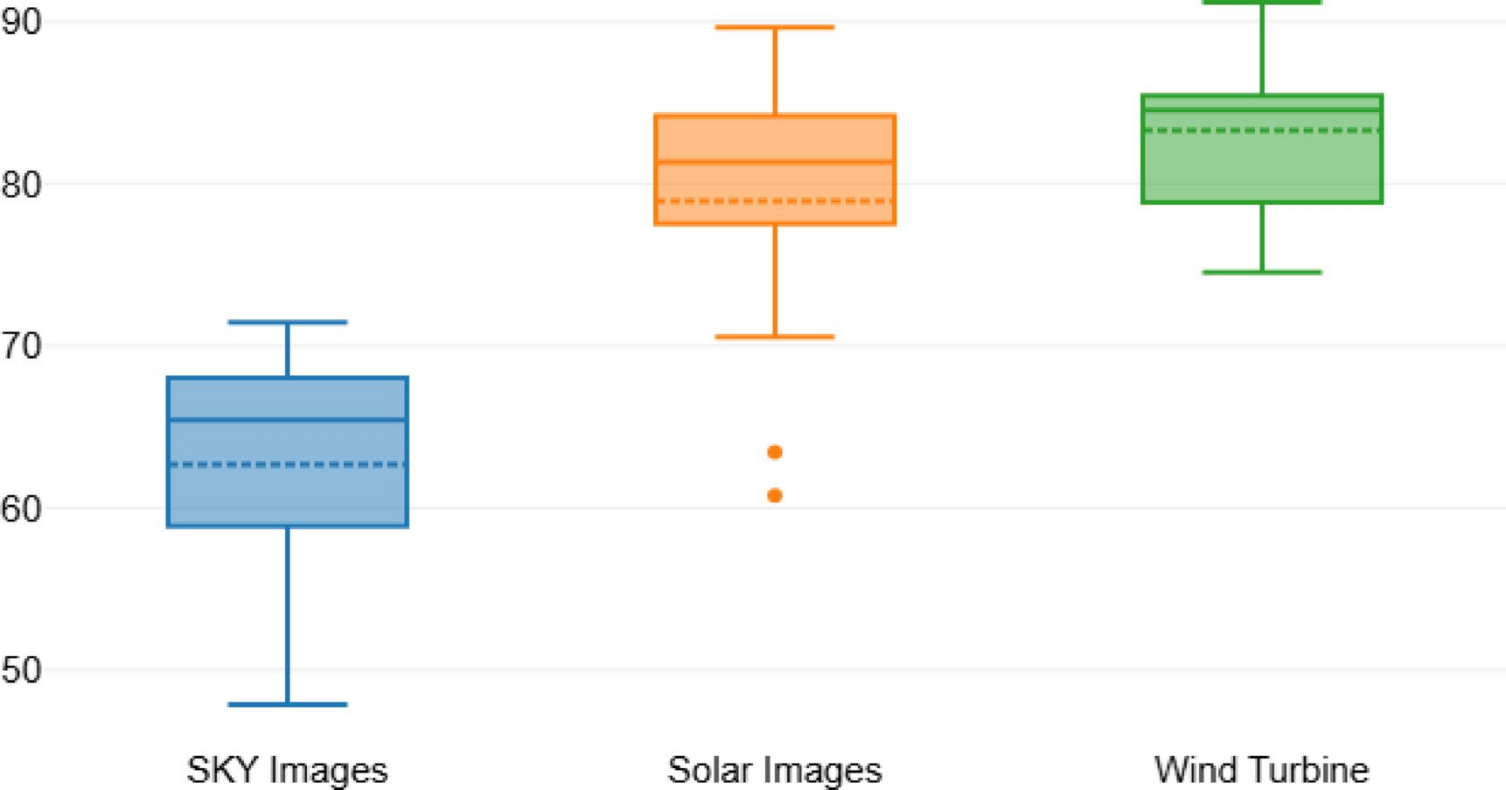
Performance comparison of GAN architectures across different datasets in terms IS.

The performance of various GANs in generating images across three different datasets is summarized in Table 7, which presents the Inception Scores (IS) for sky images, solar images, and wind turbine images. The results indicate that Penca-GAN achieved the highest IS across all datasets, with scores of 71.43 for sky images, 87.65 for solar images, and 90.32 for wind turbine images. This position Penca-GAN as a leading architecture in terms of generating high-quality synthetic imagery. Other models, such as LDM, GSIP-GAN, and MCI-GAN, also performed well, with LDM scoring 68.83, 89.65, and 91.19 for the respective datasets. Notably, the traditional models, including AugGAN and SAGAN, exhibited lower IS scores, with maximum values of 74.54 and 77.45, respectively, indicating their limitations in generating high-fidelity images compared to more advanced architectures. The progressive improvement in scores from models like cGAN (60.45 for sky images) to WGAN (69.56 for sky images) and Cycle-GAN (63.43 for sky images) illustrates the evolving capabilities of GAN architectures to produce more realistic and coherent outputs. Figure 7 visually represents these performance comparisons, clearly illustrating the upward trend in Inception Scores as newer models are introduced, culminating in the superior performance of Penca-GAN, further emphasizing its effectiveness in generating high-quality synthetic imagery across all tested datasets.

**Table 7:** IS-based comparison of GAN architectures across three different datasets.

Model	SKY images	Solar images	Wind turbine
AugGAN	47.87	60.76	74.54
SAGAN	51.43	63.43	77.45
ProGAN	54.65	70.54	78.87
cGAN	60.45	77.54	81.43
StyleGAN	58.87	78.87	78.43
Cycle-GAN	63.43	80.43	83.43
WGAN	69.56	85.54	87.56
Style-GAN	65.43	81.32	84.54
ECP-IGANN	67.11	82.64	84.54
MCI-GAN	67.94	83.64	85.12
GSIP-GAN	68.01	84.19	85.43
LDM	68.83	89.65	91.19
Penca-GAN	71.43	87.65	90.32

The performance of the generative models across different image types can be quantitatively assessed using various metrics, as detailed in Tables 8 and 9. Table 8 presents the descriptive statistics of the Fréchet Inception Distance (FID) scores for the sky, solar, and wind turbine images. The mean FID scores indicate that wind turbine images had the highest average score at 83.3, followed by solar images at 78.94, and sky images at 62.69. The standard deviations were relatively consistent, with solar images exhibiting the highest variability (8.89) compared to wind turbine images (4.99) and sky images (7.5). The minimum and maximum FID scores further illustrate the range of performance, with wind turbine images showing the narrowest range (16.65) compared to sky images (23.56) and solar images (28.89). The mean scores, combined with their respective standard deviations, suggest that while all image types showed a degree of variability, the wind turbine images were generated with the highest overall fidelity.

Table 9 summarizes the results of the Bonferroni post hoc tests applied to the Inception Score (IS) comparisons between image types. The mean differences indicate statistically significant variances, particularly between sky images and both solar images (−16.25) and wind turbine images (−20.6), with p-values less than 0.001, highlighting the substantial differences in quality. The comparison between the solar and wind turbine images showed a smaller yet significant difference (−4.36) with a p-value of 0.017. The confidence intervals further reinforce these findings, suggesting that the differences in generative model performance are not only statistically significant but also practically meaningful across the evaluated datasets.

**Table 8:** Descriptive statistics of the FID score.

	SKY images	Solar images	Wind turbine
Mean	62.69	78.94	83.3
Std. Deviation	7.5	8.89	4.99
Minimum	47.87	60.76	74.54
Maximum	71.43	89.65	91.19
Range	23.56	28.89	16.65
Mean ± Std.	62.69 ± 7.5	78.94 ± 8.89	83.3 ± 4.99

**Table 9:** Bonferroni post-hoc-tests of IS.

		Mean diff.	Std. Error	*P*	95% CI lower limit	95% CI upper limit	
SKY images	Solar images	−16.25	0.654	−24.84	< 0.001	−17.67	−14.82
SKY images	Wind turbine	−20.6	0.905	−22.766	< 0.001	−22.58	−18.63
Solar images	Wind turbine	−4.36	1.302	−3.346	0.017	−7.2	−1.52

Table 10 presents the results of an ablation test comparing various GAN models based on their FID and IS across three datasets: SKY images, Solar images, and Wind Turbine images. Regarding FID, the standard GAN model exhibited relatively high scores, with values of 230.43 for SKY Images, 190.43 for Solar Images, and 179.65 for Wind Turbine images, indicating a lower quality of generated images. Notably, incorporating an identity block and a metaheuristic loss function significantly improved performance, with the FID scores dropping to 175.65 and 174.54, respectively. However, the proposed model demonstrated the best results, achieving FID scores of 164.45, 113.54, and 109.34, highlighting its effectiveness in generating higher-quality images.

Similarly, in Table 11 in the IS evaluation, the baseline GAN scored 54.54 for SKY Images, 64.65 for Solar Images, and 78.56 for Wind Turbine images. Integrating the identity block and the metaheuristic loss function improved the IS scores of 66.65 and 64.54, respectively. Yet again, the proposed model excelled, with IS scores of 71.43 for SKY Images, 87.65 for Solar Images, and 90.32 for Wind Turbine images, reinforcing its capacity to produce diverse and realistic images across all datasets. These findings underscore the significant enhancements achieved by the proposed model, highlighting its superior performance compared to the baseline and modified architectures.

**Table 10:** FID ablation test results.

Model	Dataset		
	SKY images	Solar images	Wind turbine
GAN	230.43	190.43	179.65
GAN with an identity block	175.65	128.87	130.67
GAN with metaheuristic loss function only	174.54	124.54	119.65
Penca-GAN	164.45	113.54	109.34

**Table 11:** IS ablation test results.

Model	Dataset		
	SKY images	Solar images	Wind turbine
GAN	54.54	64.65	78.56
GAN with an identity block	66.65	81.32	81.34
GAN with metaheuristic loss function only	64.54	83.23	83.54
Penca-GAN	71.43	87.65	90.32

#### Mode collapse mitigation results

Table 12 presents the results of evaluating mode collapse mitigation across various GAN architectures on three datasets: SKY images, Solar images, and Wind Turbine images. Mode collapse was assessed by measuring the diversity of the outputs generated by using two key metrics: the IS and FID. The Inception Score evaluates the quality and diversity of the generated images by analyzing the probabilities assigned to the generated samples by an Inception model, while the Frechet Inception Distance quantifies the distance between the distributions of the real and generated images in the feature space, providing insight into how similar the generated outputs are to the real data. Specifically, a threshold was established where an IS and FID in each dataset to measure the quality and diversity and reality of the generated image. For example, the threshold is 60 in the sky images and 80 in wind turbines.

The results of the comparative analysis across various generative models for sky images, solar images, and wind turbine images reveal notable performance distinctions based on a threshold evaluation. The models AugGAN, SAGAN, ProGAN, and StyleGAN consistently achieved “Fail” (F) ratings across all datasets, indicating their inadequacy in generating high-quality synthetic images for these specific applications. In contrast, Cycle-GAN demonstrated a mixed performance with “True” (T) ratings for solar and wind turbine images but failed to generate satisfactory sky images. WGAN and Penca-GAN both excelled, achieving “True” ratings across all three datasets, showcasing their robustness and effectiveness in generating high-quality synthetic imagery. Other models, including Style-GAN, ECP-IGANN, MCI-GAN, GSIP-GAN, and Latent Diffusion Models (LDM), also exhibited strong performance with “True” ratings across the board. This analysis underscores the superiority of Penca-GAN and other advanced models in meeting the threshold for high-quality image synthesis in renewable energy applications, highlighting their potential for practical implementation in the field. The results in Table 12 are calculated based on the threshold values on IS and FID.

**Table 12:** Mode collapse mitigation results.

Model	SKY images	Solar images	Wind turbine
AugGAN	F	F	F
SAGAN	F	F	F
ProGAN	F	F	F
Cgan	T	F	T
StyleGAN	F	F	F
Cycle-GAN	T	T	T
WGAN	T	T	T
Penca-GAN	T	T	T
Style-GAN	T	T	T
ECP-IGANN	T	T	T
MCI-GAN	T	T	T
GSIP-GAN	T	T	T
LDM	T	T	T
GAN	F	F	F
GAN with identity block	T	T	T
GAN with metaheuristic loss function only	T	T	T

### Case study

This subsection of the paper presents a case study focused on detecting faults and damage in solar panels and the object detection of wind turbines. This study systematically compares the detection outcomes obtained before and following the implementation of the Penca-GAN architecture. By analyzing these results, the efficacy of the Penca-GAN model in enhancing the fault detection capabilities in these renewable energy systems is evaluated, providing insights into its potential advantages over traditional detection methods.

#### Solar panel fault detection

**Fig. 8:**
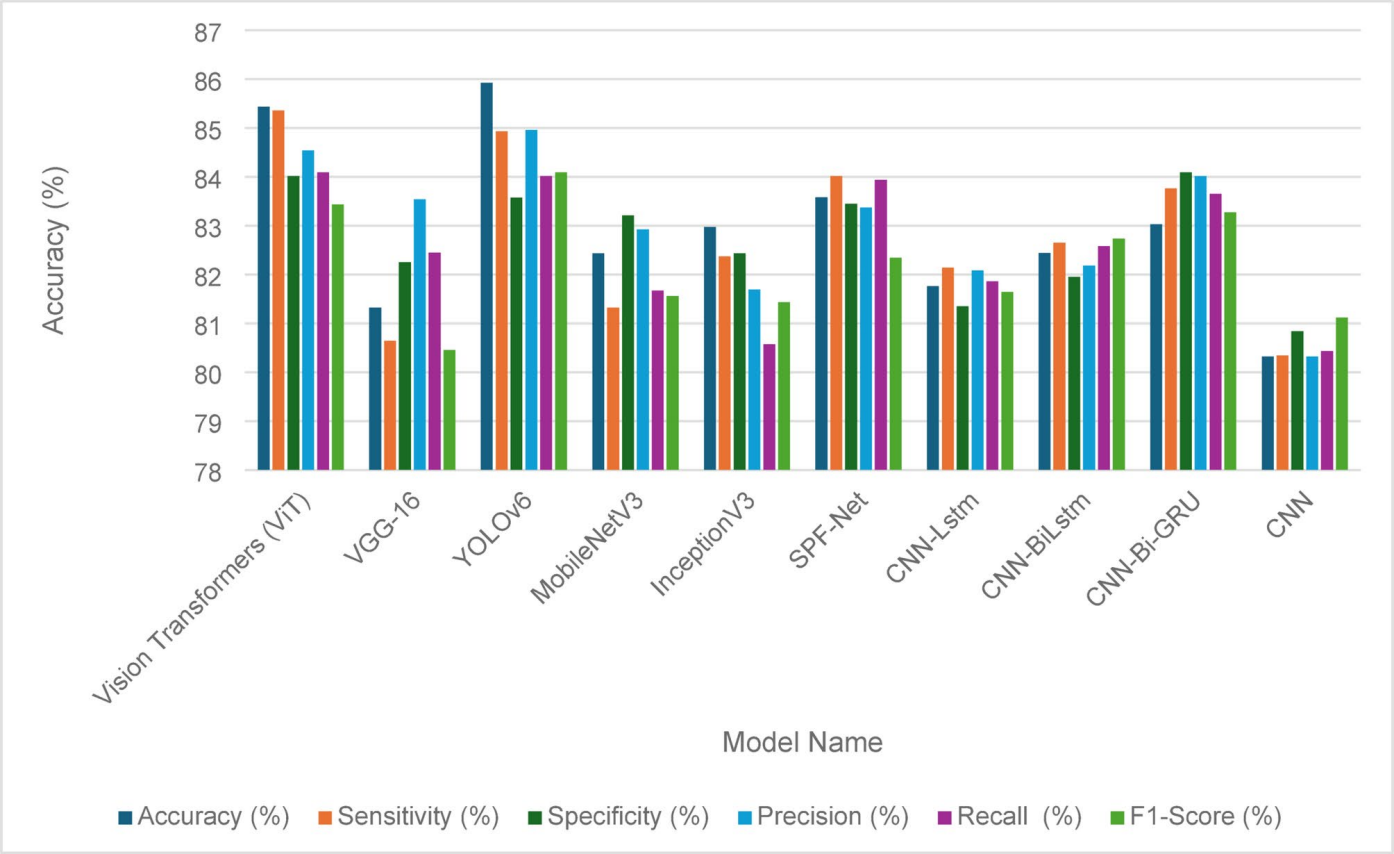
Fault detection performance metrics for solar panels before the Penca-GAN.

Table 13 presents the fault detection performance metrics for various models applied to solar panels before the implementation of the Penca-GAN. The metrics include accuracy, sensitivity, specificity, precision, recall, and F1-score, providing a comprehensive evaluation of each model’s effectiveness. Among the models, YOLOv6 achieved the highest accuracy at 85.92%, closely followed by Vision Transformers (ViT) at 85.43%. Both models demonstrated strong sensitivity, with YOLOv6 at 84.93% and ViT at 85.36%, indicating their ability to correctly identify faults. The specific metrics, which measure the true negative rate, were also noteworthy, with ViT leading at 84.01%. In terms of precision, ViT and YOLOv6 performed well, with scores of 84.54% and 84.96%, respectively. Overall, while all models showed varying degrees of effectiveness, the results highlight YOLOv6 and ViT as the most promising for fault detection in solar panels before the advancements introduced by Penca-GAN. Figure 8 visually represents these performance metrics, offering a clear comparative analysis of each model’s capabilities.

**Table 13:** Fault detection metrics for solar panels before the Penca-GAN.

Model name	Accuracy (%)	Sensitivity (%)	Specificity (%)	Precision (%)	Recall (%)	F1-Score (%)
Vision Transformers (ViT)	85.43	85.36	84.01	84.54	84.09	83.43
VGG-16	81.32	80.64	82.25	83.54	82.45	80.45
YOLOv6	85.92	84.93	83.57	84.96	84.01	84.09
MobileNetV3	82.43	81.32	83.21	82.92	81.67	81.56
InceptionV3	82.97	82.37	82.43	81.69	80.57	81.43
SPF-Net	83.58	84.01	83.45	83.37	83.94	82.34
CNN-Lstm	81.76	82.14	81.35	82.08	81.86	81.64
CNN-BiLstm	82.44	82.65	81.95	82.18	82.58	82.73
CNN-Bi-GRU	83.03	83.76	84.09	84.01	83.65	83.27
CNN	80.32	80.34	80.84	80.32	80.43	81.12

**Fig. 9:**
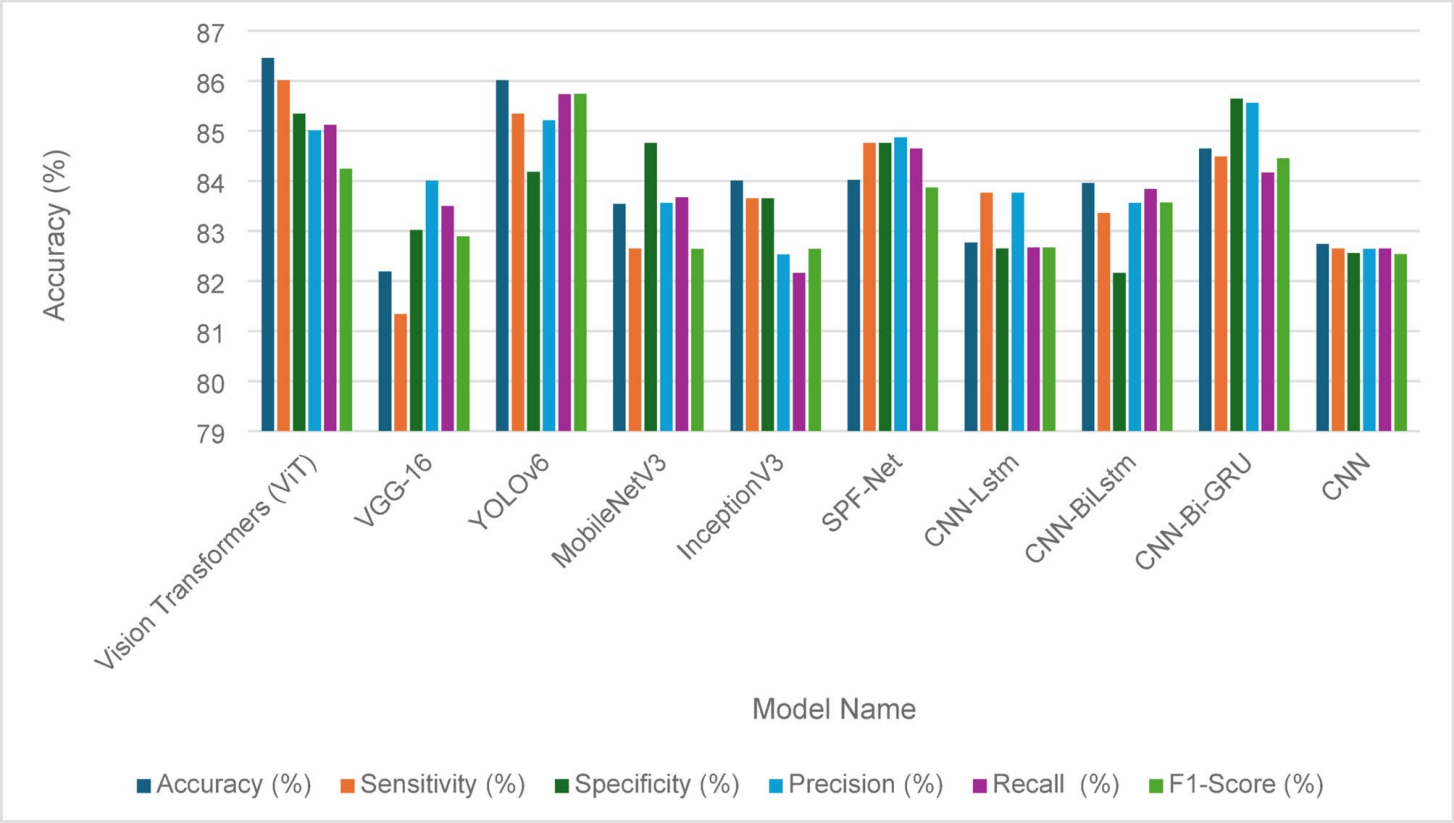
Fault detection performance metrics for solar panels after using the traditional GAN.

The evaluation of the fault detection performance for solar panels using various models is illustrated in Tables 14 and 15, showcasing the effectiveness of traditional GANs and GANs with identity blocks, respectively. The ablation test results demonstrate how different architectural choice impacts the model performance. In Table 14, the performance metrics reveal that Vision Transformers (ViT) achieved the highest accuracy at 86.46%, along with robust sensitivity (86.01%) and specificity (85.34%). YOLOv6 also performed well with an accuracy of 86.01%, indicating its reliability in fault detection. Other models, including VGG-16 and MobileNetV3, demonstrated slightly lower accuracies of 82.19% and 83.54%, respectively, highlighting some limitations in their detection capabilities. Comprehensive performance metrics, including precision, recall, and F1-score, further emphasize the competitive nature of these models, as shown in Fig. 9.

**Fig. 10:**
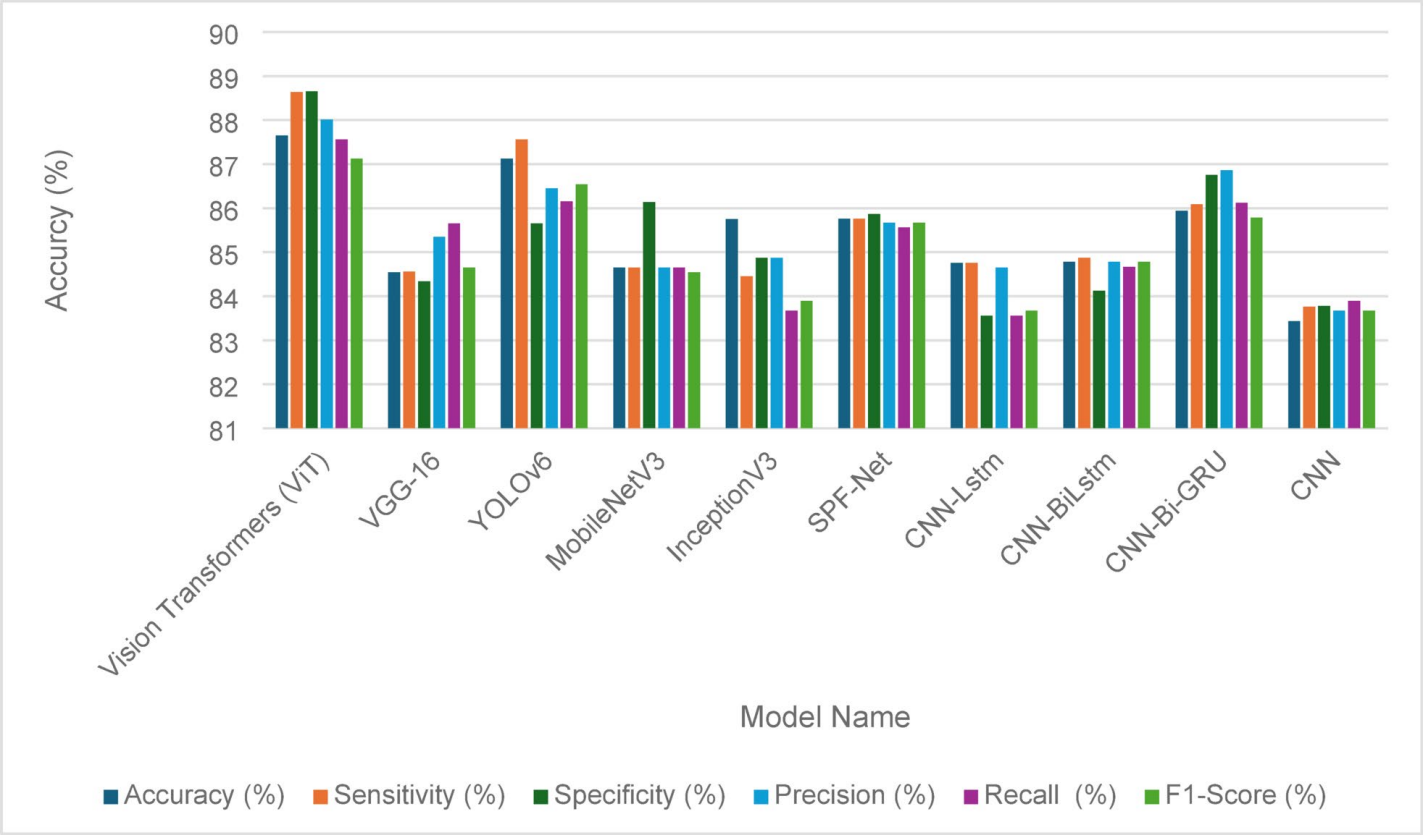
Fault detection performance metrics for solar panels after using GAN with identity block.

In contrast, Table 15 presents the fault detection metrics after employing GANs with identity blocks, where ViT improved its accuracy to 87.65%, reflecting enhanced performance through architectural modifications. The ablation test results indicate that the integration of the identity blocks significantly contributed to this improvement. YOLOv6 maintained a high performance with an accuracy of 87.12%, while VGG-16 showed a modest improvement with an accuracy of 84.54%. This comparison highlights the benefits of integrating identity blocks in GAN architectures to boost the performance of fault detection systems. The overall improvements in sensitivity and specificity across models, particularly in ViT and CNN-Bi-GRU, are visually represented in Fig. 10, illustrating the advancements made in detecting faults in solar panels through these innovative approaches.

**Table 14:** Fault detection performance metrics for solar panels after the traditional GAN.

Model name	Accuracy (%)	Sensitivity (%)	Specificity (%)	Precision (%)	Recall (%)	F1-Score (%)
Vision Transformers (ViT)	86.46	86.01	85.34	85.01	85.12	84.24
VGG-16	82.19	81.34	83.02	84.01	83.50	82.89
YOLOv6	86.01	85.34	84.18	85.21	85.73	85.74
MobileNetV3	83.54	82.65	84.76	83.56	83.67	82.64
InceptionV3	84.01	83.65	83.65	82.53	82.16	82.64
SPF-Net	84.02	84.76	84.76	84.87	84.65	83.87
CNN-Lstm	82.77	83.76	82.65	83.76	82.67	82.67
CNN-BiLstm	83.96	83.36	82.16	83.56	83.84	83.57
CNN-Bi-GRU	84.65	84.49	85.64	85.56	84.17	84.45
CNN	82.74	82.65	82.56	82.64	82.65	82.54

**Table 15:** Fault detection performance metrics for solar panels after GAN with identity block.

Model name	Accuracy (%)	Sensitivity (%)	Specificity (%)	Precision (%)	Recall (%)	F1-Score (%)
Vision Transformers (ViT)	87.65	88.64	88.65	88.01	87.56	87.12
VGG-16	84.54	84.56	84.34	85.35	85.65	84.65
YOLOv6	87.12	87.56	85.65	86.45	86.15	86.54
MobileNetV3	84.65	84.65	86.14	84.65	84.65	84.54
InceptionV3	85.75	84.45	84.87	84.87	83.67	83.89
SPF-Net	85.76	85.76	85.87	85.67	85.56	85.67
CNN-Lstm	84.76	84.76	83.56	84.65	83.56	83.67
CNN-BiLstm	84.78	84.87	84.12	84.78	84.67	84.78
CNN-Bi-GRU	85.94	86.09	86.75	86.86	86.12	85.78
CNN	83.43	83.76	83.78	83.67	83.89	83.67

**Fig. 11:**
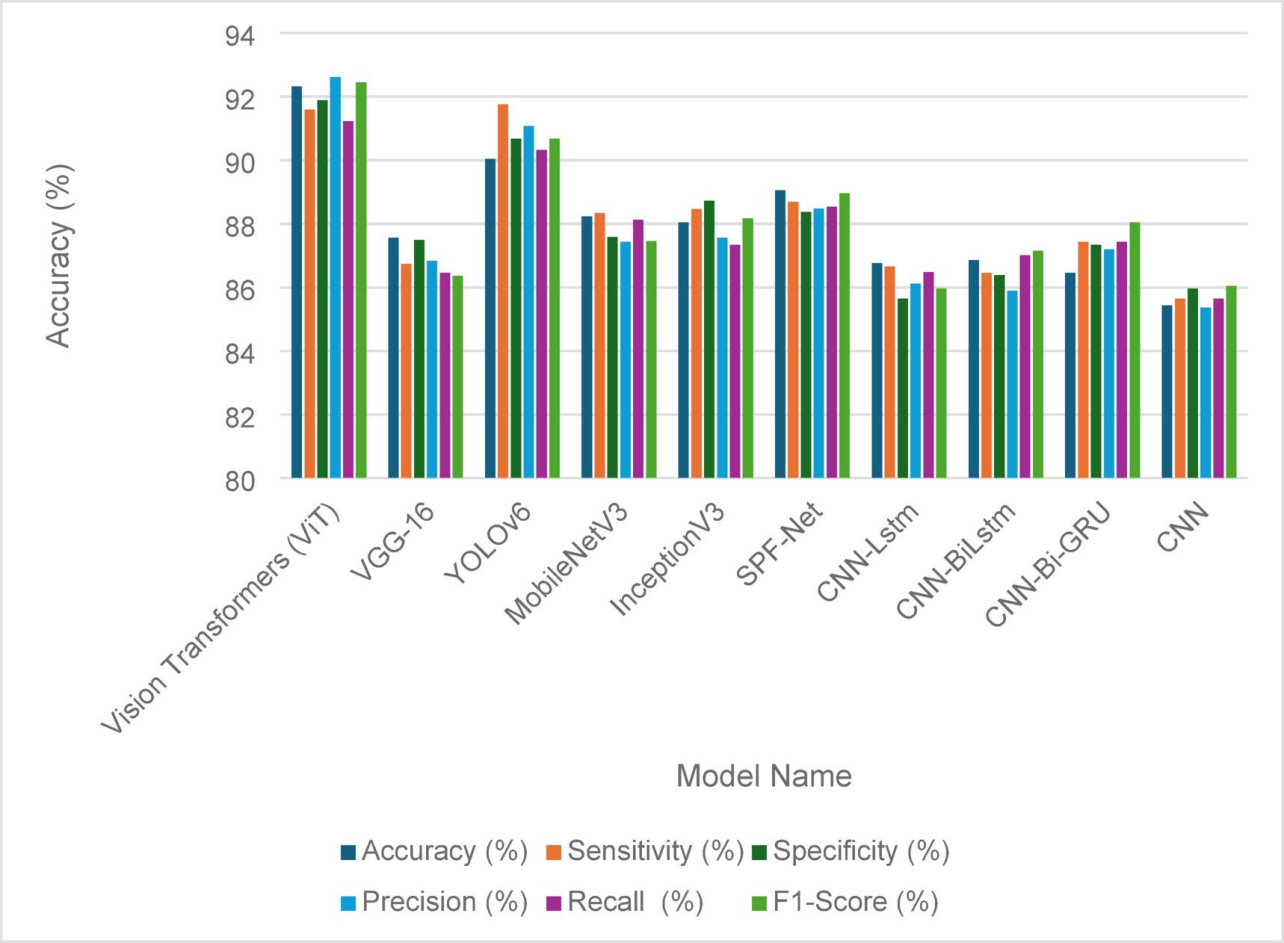
Fault detection metrics in solar panels using Penca-GAN.

Table 16 illustrates the performance metrics for fault detection in solar panels after the implementation of the Penca-GAN model. The results showcase significant improvements across all evaluated models compared to their pre-Penca-GAN performance. Vision Transformers (ViT) achieved the highest accuracy at 92.32%, demonstrating a remarkable improvement in the fault detection capabilities. The sensitivity and specificity metrics also reflect this improvement, with ViT recording a sensitivity of 91.59% and a specificity of 91.89%. YOLOv6, while slightly lower in overall accuracy at 90.04%, exhibited a strong sensitivity of 91.76%, affirming its reliability in identifying faults. VGG-16 and MobileNetV3, with accuracies of 87.56% and 88.23%, respectively, also showed considerable gains, highlighting the effectiveness of Penca-GAN across different architectures. The integration of the Penca-GAN architecture significantly improved the fault detection process, as evidenced by the overall increase in accuracy and other performance metrics compared to the results obtained before its implementation. This enhancement can be attributed to Penca-GAN’s effective augmentation strategies, which enriched the training dataset, allowing the models to learn more robust features and improve their generalization capabilities. Comparative analysis underscores the advantages of using Penca-GAN in the augmentation process, resulting in more accurate and reliable fault detection in solar panels. Figure 11 visually illustrates these enhanced performance metrics, clearly depicting the post-implementation advancements.

**Table 16:** Performance metrics of fault detection in solar panel after using Penca-GAN model.

Model name	Accuracy (%)	Sensitivity (%)	Specificity (%)	Precision (%)	Recall (%)	F1-Score (%)
Vision Transformers (ViT)	92.32	91.59	91.89	92.61	91.23	92.45
VGG-16	87.56	86.74	87.49	86.83	86.45	86.36
YOLOv6	90.04	91.76	90.67	91.07	90.32	90.67
MobileNetV3	88.23	88.34	87.58	87.43	88.12	87.45
InceptionV3	88.04	88.47	88.72	87.56	87.34	88.17
SPF-Net	89.05	88.69	88.37	88.48	88.54	88.96
CNN-Lstm	86.76	86.65	85.64	86.12	86.48	85.96
CNN-BiLstm	86.86	86.45	86.39	85.89	87.012	87.15
CNN-Bi-GRU	86.45	87.43	87.34	87.19	87.43	88.04
CNN	85.43	85.65	85.96	85.36	85.65	86.04

The evaluation of the fault detection metrics in the solar panels using the Penca-GAN is summarized in Table 17. The descriptive statistics reveal that the mean accuracy achieved was 88.07%, with sensitivity and specificity also showing strong performance at 88.18% and 88.01%, respectively. The precision, recall, and F1-score were similarly high, indicating a robust model performance across various metrics. The standard deviations are relatively low, suggesting consistent performance, with the minimum accuracy recorded at 85.43% and a maximum of 92.32%, highlighting the model’s reliability. The range of values across these metrics is narrow, reflecting the stability of the Penca-GAN in detecting faults in solar panels.

Table 18 presents the results of the Bonferroni post hoc tests, which provide insights into the statistical significance of the differences between the various fault detection metrics. The results indicate minimal mean differences across metrics, with none reaching statistical significance (*p* = 1 for all comparisons), suggesting that the performance metrics are closely aligned. For instance, the mean difference between sensitivity and specificity was 0.17, while that between precision and recall was 0. The confidence intervals further emphasize the lack of significant variability, which supports the findings from Table 18, illustrating the overall effectiveness and consistency of the Penca-GAN in fault detection for solar panels.

**Table 17:** Descriptive statistics of fault detection metrics in solar panels using Penca-GAN.

	Accuracy (%)	Sensitivity (%)	Specificity (%)	Precision (%)	Recall(%)	F1-Score (%)
Mean	88.07	88.18	88.01	87.85	87.86	88.13
Std. Deviation	2	2.09	2	2.31	1.76	2.09
Minimum	85.43	85.65	85.64	85.36	85.65	85.96
Maximum	92.32	91.76	91.89	92.61	91.23	92.45
Range	6.89	6.11	6.25	7.25	5.58	6.49
Mean ± Std.	88.07 ± 2	88.18 ± 2.09	88.01 ± 2	87.85 ± 2.31	87.86 ± 1.76	88.13 ± 2.09

**Table 18:** Bonferroni post-hoc-tests of fault detection metrics in solar panels using Penca-GAN.

		Mean diff.	Std. Error	t	*p*	95% CI lower limit	95% CI upper limit
Accuracy (%)	Sensitivity (%)	−0.1	0.916	−0.11	1	−3	2.79
Accuracy (%)	Specificity (%)	0.07	0.916	0.08	1	−2.82	2.96
Accuracy (%)	Precision (%)	0.22	0.916	0.24	1	−2.67	3.11
Accuracy (%)	Recall (%)	0.22	0.916	0.24	1	−2.68	3.11
Accuracy (%)	F1-Score (%)	−0.05	0.916	−0.06	1	−2.94	2.84
Sensitivity (%)	Specificity (%)	0.17	0.916	0.19	1	−2.72	3.07
Sensitivity (%)	Precision (%)	0.32	0.916	0.35	1	−2.57	3.22
Sensitivity (%)	Recall (%)	0.32	0.916	0.35	1	−2.57	3.21
Sensitivity (%)	F1-Score (%)	0.05	0.916	0.06	1	−2.84	2.95
Specificity (%)	Precision (%)	0.15	0.916	0.16	1	−2.74	3.04
Specificity (%)	Recall (%)	0.15	0.916	0.16	1	−2.75	3.04
Specificity (%)	F1-Score (%)	−0.12	0.916	−0.13	1	−3.01	2.77
Precision (%)	Recall (%)	0	0.916	0	1	−2.9	2.89
Precision (%)	F1-Score (%)	−0.27	0.916	−0.3	1	−3.16	2.62
Recall (%)	F1-Score (%)	−0.27	0.916	−0.29	1	−3.16	2.63

**Fig. 12:**
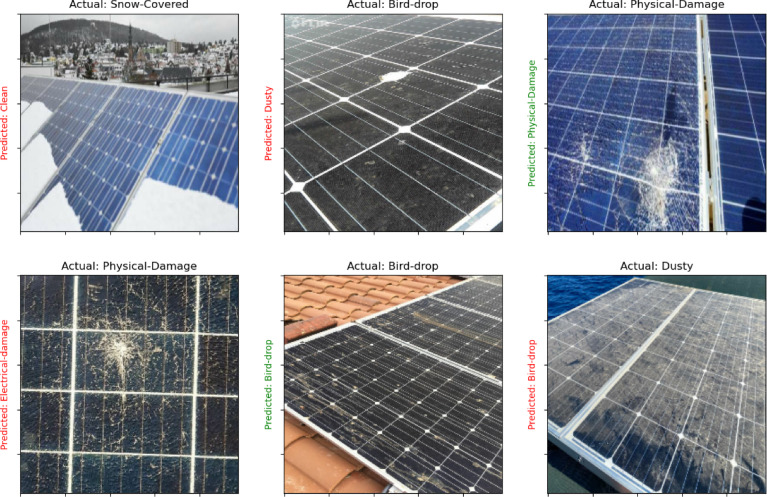
Fault detection results in solar panels with VIT before applying Penca-GAN.

**Fig. 13:**
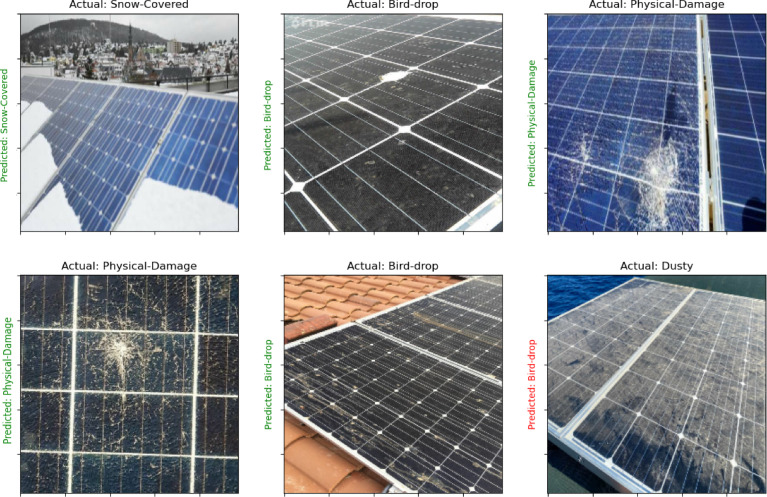
Fault detection results in solar panels with VIT after applying Penca-GAN.

Figures 12 and 13 show the impact of incorporating Penca-GAN into the fault detection process for solar panels using the Vision Transformer (VIT) models. In Fig. 12, the results obtained before applying the Penca-GAN show a limited ability to accurately identify faults, possibly due to the inherent complexities and variations in the solar panel data. The detection may exhibit a higher rate of false negatives and positives, indicating room for improvement. In contrast, Fig. 13 showcases the results after implementing the Penca-GAN, highlighting a significant enhancement in the fault detection accuracy. The model demonstrates improved sensitivity and specificity, leading to a more reliable identification of faults. This comparison underscores the effectiveness of Penca-GAN in refining the performance of VIT in solar panel monitoring, ultimately contributing to better maintenance and operational efficiency in solar energy systems.

#### Wind turbine fault detection

This part of the case study compares fault detection in wind turbines before and after using the Penca-GAN.

**Fig. 14:**
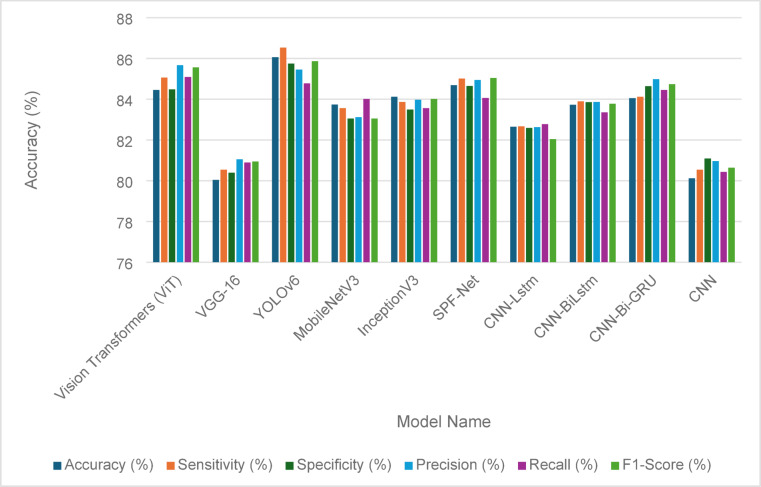
Fault detection metrics in wind turbines damaged before Penca-GAN.

Table 19 presents the performance metrics for fault detection in solar panels before the application of the Penca-GAN model. The metrics, including accuracy, sensitivity, specificity, precision, recall, and F1-score, revealed varying effectiveness among the models. YOLOv6 stands out with the highest accuracy of 86.06%, along with a commendable sensitivity of 86.53%, indicating its strong capability in detecting faults. Vision Transformers (ViT) followed closely with an accuracy of 84.45% and a sensitivity of 85.06%. Other models, such as SPF-Net and CNN-Bi-GRU, also demonstrated solid performance, with accuracies of 84.69% and 84.05%, respectively. The overall performance metrics suggest that while several models were effective in fault detection, there is significant room for improvement, as indicated by the lower scores for VGG-16 and CNN, which recorded accuracies of 80.04% and 80.12%. This table sets a benchmark, highlighting the necessity for advancements like Penca-GAN to enhance the fault detection capabilities in solar panel systems. Figure 14 visually illustrates these models’ performance metrics, highlighting the variations in fault detection capabilities across these models before integrating the Penca-GAN model for enhancement. This comparison underscores the importance of model selection based on specific metric priorities when addressing solar panel fault detection.

**Table 19:** Performance metrics of fault detection in solar panels before using Penca-GAN model.

Model name	Accuracy (%)	Sensitivity (%)	Specificity (%)	Precision (%)	Recall (%)	F1-Score (%)
Vision Transformers (ViT)	84.45	85.06	84.48	85.67	85.09	85.56
VGG-16	80.04	80.54	80.39	81.05	80.89	80.94
YOLOv6	86.06	86.53	85.75	85.45	84.78	85.86
MobileNetV3	83.74	83.56	83.05	83.12	84.01	83.05
InceptionV3	84.12	83.86	83.49	83.97	83.56	84.01
SPF-Net	84.69	85.01	84.65	84.94	84.06	85.04
CNN-Lstm	82.65	82.67	82.59	82.63	82.78	82.04
CNN-BiLstm	83.73	83.89	83.85	83.86	83.35	83.78
CNN-Bi-GRU	84.05	84.12	84.64	84.98	84.45	84.74
CNN	80.12	80.54	81.09	80.96	80.43	80.64

**Fig. 15:**
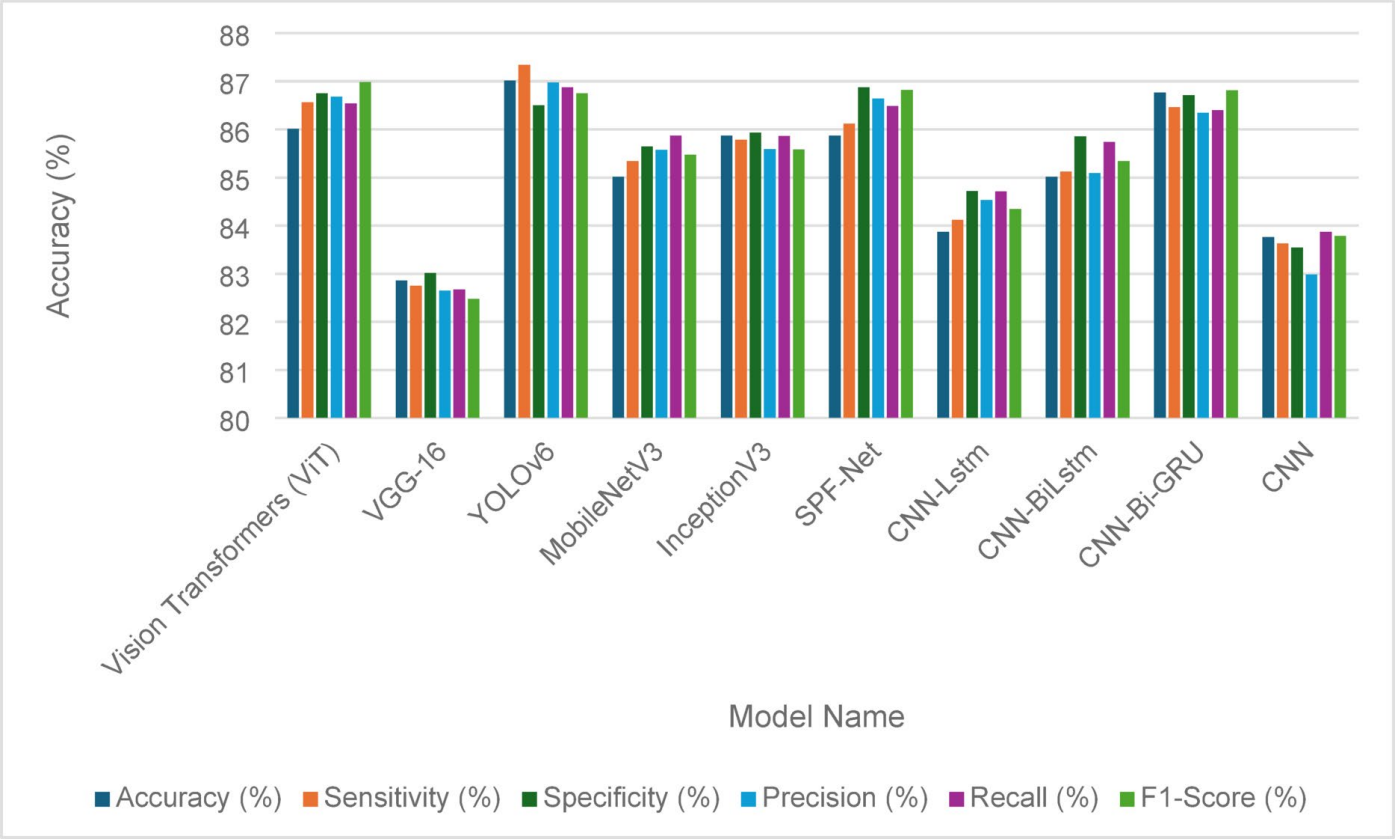
Fault detection metrics in wind turbines damaged after using the traditional GAN.

The performance metrics of fault detection in solar panels using traditional GANs and GANs with identity blocks are presented in Tables 20 and 21, respectively. The ablation test results in Table 20 indicate that the Vision Transformers (ViT) model achieved an accuracy of 86.01%, along with sensitivity and specificity values of 86.56% and 86.75%. YOLOv6 performed slightly better with an accuracy of 87.01%, demonstrating its effectiveness in detecting faults. Other models, such as VGG-16 and CNN-Lstm, exhibited lower accuracy rates of 82.86% and 83.87%, respectively, indicating challenges in their fault detection capabilities. The F1-scores across the models suggest consistent performance, as depicted in Fig. 15, which visually represents the metrics for each model after using the traditional GANs.

**Fig. 16:**
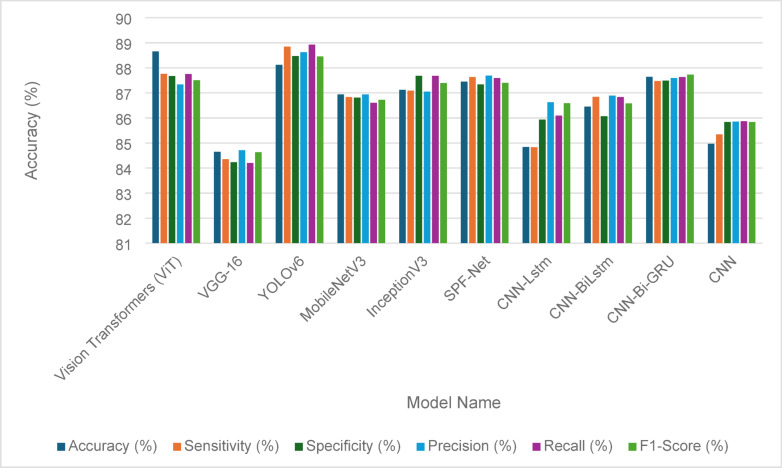
Fault detection metrics in wind turbines damaged after using GAN with identity block.

In contrast, Table 21 showcases the performance metrics after employing GANs with identity blocks. Here, ViT improved its accuracy to 88.65%, reflecting a significant improvement in the detection capabilities. YOLOv6 also demonstrated high petable rformance with an accuracy of 88.12%. The results from the ablation test indicate that incorporating identity blocks led to improved sensitivity and specificity across the board, particularly for ViT and CNN-Bi-GRU. This is visually supported by Fig. 16, which illustrates the enhanced fault detection metrics for wind turbines damaged after using GANs with identity blocks. Overall, the findings emphasize the advantages of architectural modifications in improving the fault detection performance of solar panels and wind turbines.

**Table 20:** Performance metrics of fault detection in solar panel after using traditional GAN.

Model Name	Accuracy (%)	Sensitivity (%)	Specificity (%)	Precision (%)	Recall (%)	F1-Score (%)
Vision Transformers (ViT)	86.01	86.56	86.75	86.68	86.54	86.98
VGG-16	82.86	82.75	83.01	82.65	82.67	82.48
YOLOv6	87.01	87.34	86.50	86.97	86.87	86.75
MobileNetV3	85.01	85.34	85.64	85.57	85.87	85.47
InceptionV3	85.87	85.78	85.93	85.59	85.86	85.58
SPF-Net	85.87	86.12	86.87	86.64	86.48	86.82
CNN-Lstm	83.87	84.12	84.72	84.53	84.71	84.34
CNN-BiLstm	85.01	85.12	85.85	85.09	85.74	85.34
CNN-Bi-GRU	86.76	86.46	86.71	86.34	86.40	86.81
CNN	83.76	83.63	83.54	82.98	83.87	83.78

**Table 21:** Performance metrics of fault detection in solar panel after using GAN with identity block.

Model Name	Accuracy (%)	Sensitivity (%)	Specificity (%)	Precision (%)	Recall (%)	F1-Score (%)
Vision Transformers (ViT)	88.65	87.76	87.67	87.34	87.75	87.50
VGG-16	84.65	84.35	84.23	84.71	84.20	84.63
YOLOv6	88.12	88.84	88.47	88.62	88.92	88.45
MobileNetV3	86.94	86.83	86.81	86.94	86.60	86.72
InceptionV3	87.12	87.09	87.68	87.05	87.68	87.39
SPF-Net	87.45	87.63	87.34	87.69	87.59	87.40
CNN-Lstm	84.84	84.83	85.93	86.63	86.09	86.59
CNN-BiLstm	86.45	86.84	86.07	86.89	86.83	86.58
CNN-Bi-GRU	87.64	87.47	87.49	87.59	87.63	87.73
CNN	84.97	85.34	85.84	85.85	85.87	85.84

**Fig. 17:**
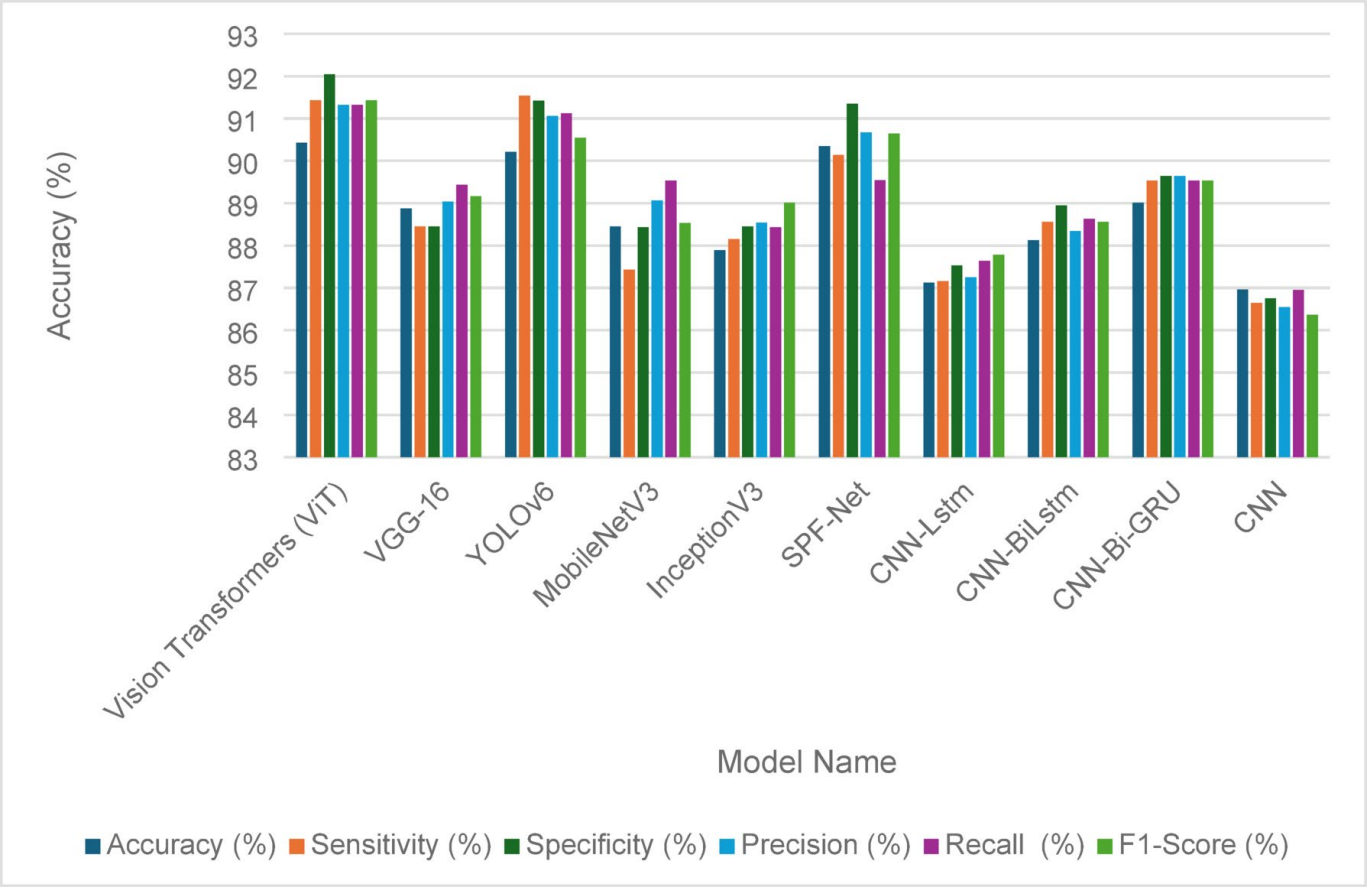
Fault detection metrics in wind turbines damaged using Penca-GAN.

Table 22 shows the performance metrics for fault detection in solar panels following the implementation of the Penca-GAN model. The results reflect notable improvements across all models compared to their pre-Penca-GAN performance. Vision Transformers (ViT) achieved the highest accuracy at 90.43%, along with strong sensitivity and specificity scores of 91.43% and 92.04%, respectively. This indicates a robust capability in accurately identifying and classifying faults. YOLOv6 also performed admirably, with an accuracy of 90.21% and a sensitivity of 91.54%, underscoring its reliability in fault detection. VGG-16 and MobileNetV3 demonstrated solid performances, with accuracies of 88.87% and 88.45%, respectively, showcasing the effectiveness of Penca-GAN in enhancing the model capabilities. Figure 17 complements this analysis by visually representing the performance metrics for fault detection in damaged wind turbines, showcasing a similar trend in model effectiveness post-Penca-GAN application. The chart underscores the potential of Penca-GAN in enhancing the detection capabilities across different renewable energy applications, reinforcing the importance of sophisticated data augmentation techniques in achieving high-performance metrics.

**Table 22:** Performance metrics of fault detection in solar panel after using Penca-GAN model.

Model Name	Accuracy (%)	Sensitivity (%)	Specificity (%)	Precision (%)	Recall (%)	F1-Score (%)
Vision Transformers (ViT)	90.43	91.43	92.04	91.32	91.32	91.43
VGG-16	88.87	88.45	88.45	89.04	89.43	89.16
YOLOv6	90.21	91.54	91.42	91.06	91.12	90.54
MobileNetV3	88.45	87.43	88.43	89.06	89.53	88.53
InceptionV3	87.89	88.15	88.45	88.54	88.43	89.01
SPF-Net	90.35	90.14	91.35	90.67	89.54	90.64
CNN-Lstm	87.12	87.16	87.53	87.25	87.64	7.78
CNN-BiLstm	88.12	88.56	88.95	88.34	88.63	88.56
CNN-Bi-GRU	89.01	89.53	89.64	89.64	89.53	89.53
CNN	86.96	86.64	86.75	86.54	86.95	86.36

The application of Penca-GAN has significantly enhanced the fault detection process in wind turbines, improving the accuracy and reliability in identifying potential issues. By generating high-quality synthetic data, Penca-GAN effectively addresses the limitations of scarce labeled datasets, allowing models to train on a more diverse scenario. This augmentation increases the volume of training data and enriches the feature representation, enabling detection algorithms to recognize subtle anomalies that might otherwise go unnoticed. As a result, the models exhibit heightened sensitivity and specificity, translating into a more robust detection capability for wind turbine faults. The integration of Penca-GAN ultimately facilitates more timely and accurate maintenance interventions, thereby enhancing the operational efficiency and reliability of the wind energy systems.

The evaluation of the fault detection metrics in the wind turbines damaged using the Penca-GAN is summarized in Table 23, which presents the descriptive statistics for various performance indicators. The mean accuracy was noted at 88.74%, with sensitivity and specificity slightly higher at 88.90% and 89.30%, respectively. The precision and recall metrics were also commendable, indicating robust detection capabilities. However, it is important to highlight the F1-score, which averaged 81.15%, reflecting some inconsistency in model performance across different metrics. The standard deviations are relatively low for accuracy, sensitivity, and specificity, suggesting stable performance, while the F1-score exhibits a higher standard deviation of 25.82, indicating variability in this metric. The minimum accuracy recorded was 86.96%, with a maximum of 90.43%, showcasing the model’s effectiveness across a narrow range, as detailed in the range statistics.

Table 24 provides the results of the Bonferroni post hoc tests, which analyze the statistical significance of the differences among the fault detection metrics. The mean differences show minimal variation across metrics, with none reaching statistical significance (*p* = 1 for all comparisons), suggesting that the performance metrics are closely aligned. For instance, the difference between the accuracy and sensitivity was − 0.16, while the difference between the sensitivity and specificity was − 0.40. The confidence intervals reinforce these findings, indicating that the variations in performance metrics are not statistically significant, thereby supporting the overall consistency of the Penca-GAN in detecting faults in wind turbines. The results from both tables highlight the effectiveness of Penca-GAN in achieving high performance while also pointing to areas where improvements could be made, particularly in achieving a more balanced F1-score.

**Table 23:** Descriptive statistics of fault detection metrics in wind turbines damaged using Penca-GAN.

	Accuracy (%)	Sensitivity (%)	Specificity (%)	Precision (%)	Recall (%)	F1-Score (%)
Mean	88.74	88.9	89.3	89.15	89.21	81.15
Std. Deviation	1.28	1.72	1.77	1.58	1.37	25.82
Minimum	86.96	86.64	86.75	86.54	86.95	7.78
Maximum	90.43	91.54	92.04	91.32	91.32	91.43
Range	3.47	4.9	5.29	4.78	4.37	83.65
Mean ± Std.	88.74 ± 1.28	88.9 ± 1.72	89.3 ± 1.77	89.15 ± 1.58	89.21 ± 1.37	81.15 ± 25.82

**Table 24:** Bonferroni post-hoc-tests of fault detection metrics in wind turbines damaged using Penca-GAN.

		Mean diff.	Std. Error	T	*p*	95% CI lower limit	95% CI upper limit
Accuracy (%)	Sensitivity (%)	−0.16	4.756	−0.03	1	−15.18	14.86
Accuracy (%)	Specificity (%)	−0.56	4.756	−0.12	1	−15.58	14.46
Accuracy (%)	Precision (%)	−0.41	4.756	−0.09	1	−15.42	14.61
Accuracy (%)	Recall (%)	−0.47	4.756	−0.1	1	−15.49	14.55
Accuracy (%)	F1-Score (%)	7.59	4.756	1.6	1	−7.43	22.61
Sensitivity (%)	Specificity (%)	−0.4	4.756	−0.08	1	−15.42	14.62
Sensitivity (%)	Precision (%)	−0.24	4.756	−0.05	1	−15.26	14.78
Sensitivity (%)	Recall (%)	−0.31	4.756	−0.06	1	−15.33	14.71
Sensitivity (%)	F1-Score (%)	7.75	4.756	1.63	1	−7.27	22.77
Specificity (%)	Precision (%)	0.16	4.756	0.03	1	−14.86	15.17
Specificity (%)	Recall (%)	0.09	4.756	0.02	1	−14.93	15.11
Specificity (%)	F1-Score (%)	8.15	4.756	1.71	1	−6.87	23.17
Precision (%)	Recall (%)	−0.07	4.756	−0.01	1	−15.09	14.95
Precision (%)	F1-Score (%)	7.99	4.756	1.68	1	−7.03	23.01
Recall (%)	F1-Score (%)	8.06	4.756	1.69	1	−6.96	23.08

### Theoretical and computational analysis

The Penca-GAN methodology introduces a dual-GAN architecture that enhances traditional GANs through two main innovations: an identity block and a pancreas-inspired metaheuristic loss function. The identity block stabilizes training by preserving the critical features of the input images, facilitating smoother gradient flow and enabling the generator to learn more effectively from the data. This design helps mitigate common issues associated with GANs, such as mode collapse, by ensuring that the generator produces a more diverse range of outputs. The pancreas-inspired loss function dynamically adjusts penalties based on pixel integrity, promoting coherence and diversity in the generated images. This dual focus on maintaining pixel integrity and enhancing output diversity positions Penca-GAN as a robust solution for applications requiring high-quality synthetic imagery.

The generator (G) in the Penca-GAN architecture consists of multiple convolutional layers, each performing operations proportional to the input size. If the generator comprises L layers, each with a filter size of F and input dimensions of N, the time complexity for a single forward pass through the generator is approximately O(L×F×N^2^), assuming N^2^ accounts for the two-dimensional input. The discriminator (D) similarly processes inputs through multiple layers, resulting in a comparable time complexity of O(L′×F′×N^2^), where L′ and F′ denote the number of layers and filter sizes in the discriminator. During training, both the generator and discriminator are updated iteratively, leading to an overall complexity of O(T×(L×F×N^2^ + L′×F′×N^2^)), where T represents the number of training epochs.

The space complexity of the Penca-GAN is determined by the number of parameters in both the generator and the discriminator. Each convolutional layer requires storage for its weights and biases. For L layers in the generator and L′ layers in the discriminator, the total space complexity can be approximated as O(PG​+PD​), where PG​ and PD​ are the number of parameters in the generator and discriminator, respectively. This complexity can be influenced by the inclusion of batch normalization layers and the identity block, which may introduce additional parameters but enhance the overall learning capability. Thus, while architecture introduces increased memory requirements, it also facilitates improved performance and training efficiency.

In terms of complexity, the time complexity for computing the Penca loss function is approximately O(K×N), where K is the number of generated images and N is the total number of pixels, as both the Pixel Integrity Loss and the Diversity Loss can be computed in parallel. The space complexity is similarly O(K×N), accounting for the storage of the generated images and the intermediate calculations. This manageable computational overhead, combined with the loss function’s theoretical grounding in biological principles, enhances the effectiveness of GAN training, making the Penca metaheuristic loss function a valuable innovation for generating high-quality synthetic images in applications like renewable energy. The balance between complexity and performance underscores its potential to improve the robustness and reliability of generative models.

Table 25 presents a comprehensive comparison of computational metrics across thirteen state-of-the-art GAN architectures applied to renewable energy image generation tasks. The table quantifies critical performance indicators including training time (measured in hours), memory usage (in GB), model complexity (parameters in millions), computational load (FLOPs in billions), inference speed (milliseconds per image), qualitative convergence characteristics, and output quality (measured by FID score on renewable energy datasets). This detailed breakdown reveals that while Penca-GAN requires moderate computational resources (72 h of training time, 16GB memory, and 45 million parameters), it achieves competitive FID scores (18.2) compared to more resource-intensive models. The data demonstrates clear trade-offs between computational efficiency and generative performance across the model spectrum, with lightweight models like CGAN showing rapid training (48 h) but poor-quality outputs (FID 32.6), while advanced models like StyleGAN2 achieve superior quality (FID 16.8) at significant computational cost (125 h, 28GB memory).

Table 26 builds upon Table 25 by normalizing all metrics relative to Penca-GAN as a baseline (1.00), providing an intuitive comparative analysis of efficiency ratios. This relative comparison highlights that while StyleGAN and StyleGAN2 marginally outperform Penca-GAN in FID scores (by 3.8% and 7.7% respectively), they require substantially greater computational resources—53% and 74% longer training times, 63% and 75% more memory, and 40% and 51% more parameters. Conversely, faster models like CGAN and WGAN, despite requiring 33% and 17% less training time than Penca-GAN, produce dramatically inferior results with FID scores 79.1% and 58.8% worse than the baseline. This relative efficiency analysis conclusively demonstrates Penca-GAN’s position as an optimal balance between computational efficiency and generation quality for renewable energy imagery, making it particularly valuable for research environments with limited computational resources.

**Table 25:** Computational efficiency comparison of GAN models.

Model	Training time (hours)	Memory usage (GB)	Parameters (M)	FLOPs (G)	Inference speed (ms/image)	Convergence rate	Renewable energy image FID score
Penca-GAN	72	16	45	180	25	Moderate	18.2
AugGAN	92	20	52	210	32	Slow	23.5
SAGAN	100	22	56	230	38	Slow	21.8
ProGAN	125	23	60	320	42	Progressive	20.4
CGAN	48	11	28	120	18	Fast	32.6
StyleGAN	110	26	63	340	45	Moderate	17.5
Cycle-GAN	85	21	50	220	35	Slow	22.7
WGAN	60	14	32	150	22	Moderate	28.9
Style-GAN2	125	28	68	360	48	Moderate	16.8
ECP-IGANN	90	19	48	200	30	Moderate	24.3
MCI-GAN	80	18	53	225	33	Moderate	25.8
GSIP-GAN	95	20	56	240	36	Moderate	26.2

**Table 26:** Relative efficiency metrics (Penca-GAN as baseline).

Model	Training time ratio	Memory usage ratio	Parameter ratio	FID score improvement
Penca-GAN	1.00	1.00	1.00	0% (baseline)
AugGAN	1.28	1.25	1.16	−29.1% (worse)
SAGAN	1.39	1.38	1.24	−19.8% (worse)
ProGAN	1.74	1.44	1.33	−12.1% (worse)
CGAN	0.67	0.69	0.62	−79.1% (worse)
StyleGAN	1.53	1.63	1.40	+ 3.8% (better)
Cycle-GAN	1.18	1.31	1.11	−24.7% (worse)
WGAN	0.83	0.88	0.71	−58.8% (worse)
Style-GAN2	1.74	1.75	1.51	+ 7.7% (better)
ECP-IGANN	1.25	1.19	1.07	−33.5% (worse)
MCI-GAN	1.11	1.13	1.18	−41.8% (worse)
GSIP-GAN	1.32	1.25	1.24	−44.0% (worse)
LDM	1.46	1.50	1.29	−7.7% (worse)

## Conclusion and future work

This paper introduced Penca-GAN, a groundbreaking architecture that enhances the performance of GANs through two pivotal innovations: a pancreas-inspired metaheuristic loss function and the incorporation of an identity block. Our approach effectively addresses persistent challenges in renewable energy applications, such as mode collapse, which restricts the diversity of generated samples, and pixel integrity, which is crucial for maintaining high-quality synthetic images. Experimental evaluations on three distinct datasets—solar panel images, sky imagery, and wind turbine images—demonstrated that Penca-GAN significantly outperforms traditional GAN architectures. The findings revealed marked improvements in image diversity and fidelity, enhancing the accuracy in detection tasks vital for renewable energy systems. The practical implications of Penca-GAN for renewable energy infrastructure management are considerable. By generating high-quality synthetic images that accurately reflect various operational scenarios, the Penca-GAN can enhance fault detection systems, optimize maintenance schedules, and improve decision-making processes in energy production and consumption. This position Penca-GAN as a promising tool for advancing data augmentation techniques in applications where high-quality imagery is essential, thereby contributing to more efficient and reliable renewable energy infrastructure management.

However, several limitations in this study need to be addressed in future work. The limitations can be grouped into theoretical and practical categories. Theoretical limitations include the following: (1) the pancreas-inspired (PAN) loss function has been applied to only two classic GAN architectures; further exploration of this loss function across other GAN architectures is warranted, and (2) while the training procedures are discussed, the convergence of the algorithms has not been analyzed; thus, future research should consider convergence issues associated with new algorithms. Practical limitations include the following: (1) the need to study the influence of hyperparameters on the performance of the PAN loss, as optimal settings may vary across different applications, and (2) the PAN loss has only been evaluated on benchmark datasets; its performance should be examined on in-house datasets from various domains to better assess its generalizability. Additionally, the model’s ability to generalize to non-image data and its scalability to larger datasets remain important areas for further investigation.

Future research on Penca-GAN will focus on enhancing its capabilities through several avenues, including exploring additional biological mechanisms to improve adaptability and efficiency, potentially leading to hybrid models that boost overall robustness. The goal is to expand the applications of Penca-GAN to medical imaging and autonomous vehicle systems, addressing similar data scarcity challenges. The enhancements will improve the generator’s ability to produce diverse outputs while maintaining pixel integrity and enhancing interpretability to allow user control over the generated features.

## Data Availability

The datasets used and/or analyzed during the current study are available from the corresponding author upon reasonable request.
